# AP1S3 Promotes Proliferation and Invasion of Cholangiocarcinoma Cells and Modulates HER2 Trafficking

**DOI:** 10.3390/cells15212039

**Published:** 2026-11-09

**Authors:** Chen Shen, Ronglin Xu, Hui Zhou, Yongxiang Wang, Zhongtao Liu, Yang Xin, Tiantian Wang, Heng Zou, Zijian Zhang, Yu Wen

**Affiliations:** Department of General Surgery, The Second Xiangya Hospital, Central South University, Changsha 410000, China; 8301180115@csu.edu.cn (C.S.); 248211126@csu.edu.cn (R.X.); zhouhui.17@csu.edu.cn (H.Z.); 228202102@csu.edu.cn (Y.W.); 178211082@csu.edu.cn (Z.L.); 248211120@csu.edu.cn (Y.X.); wangtt_17@csu.edu.cn (T.W.); 506828@csu.edu.cn (H.Z.)

**Keywords:** cholangiocarcinoma, psoriasis, AP1S3, HER2, HER2 trafficking, computational drug discovery

## Abstract

**Background:** Cholangiocarcinoma (CCA) is an inflammation-associated malignancy with limited benefit from HER2-targeted therapy. Whether psoriasis is causally linked to CCA and how AP1S3 contributes remain unclear. **Methods:** We performed exploratory two-sample Mendelian randomization (MR), integrated bulk and single-cell transcriptomes, and used CCA cell lines and xenografts to examine AP1S3 and HER2 trafficking. **Results:** Strict MR (8 IVs; MHC excluded) gave an OR of 1.00 (95% CI 0.83–1.21; *p* = 0.98); the relaxed-threshold estimate (OR 1.12, 1.01–1.25; *p* = 0.037) was not robust (independent OR 1.05, 0.96–1.14; *p* = 0.30). IL-17A/TNF-α increased AP1S3 in CCA cells and activated NF-κB/RELA, but direct RELA binding was not demonstrated. AP1S3 was associated with a SCEL+ squamous-like program. AP1S3 co-precipitated with HER2 via EIPDLL (residues 930–935, kinase domain) and modulated HER2 trafficking; direct binding, isoform specificity and active recycling were not established. **Conclusions** The psoriasis–CCA association is inconclusive. AP1S3 is a candidate inflammatory regulator of HER2 trafficking and requires direct binding, loss-of-function and clinical validation.

## 1. Introduction

Cholangiocarcinoma (CCA) is a highly malignant neoplasm originating from the epithelial cells of the bile ducts, characterized by insidious onset, high invasiveness, and an extremely poor prognosis, with a 5-year overall survival rate of less than 10% [1,2,3,4]. In recent years, the global incidence of CCA has shown a continuous upward trend, yet therapeutic options remain very limited [5,6,7,8,9]. A large body of epidemiological and basic research indicates that chronic local or systemic inflammation is a core driver of CCA development and progression [10,11,12]. However, how systemic inflammation reshapes the malignant phenotype of CCA cells remains incompletely understood.

Psoriasis is a common systemic immune-mediated disease with a global prevalence of approximately 2–3%, characterized by elevated levels of pro-inflammatory cytokines such as IL-17A and TNF-α [13,14,15]. Accumulating evidence suggests that patients with psoriasis have an increased risk of developing various solid tumors, including skin cancer, lymphoma, and gastrointestinal tumors [16,17]. Nevertheless, direct epidemiological estimates for biliary cancers are sparse: most cohorts report overall or gastrointestinal cancer risk rather than CCA specifically [16,17], and whether a causal relationship exists between psoriasis and CCA remains inconclusive [5,6,7]. More importantly, the molecular mechanisms by which the systemic inflammatory microenvironment remotely regulates the biological behavior of CCA cells remain a key scientific question to be addressed in the field.

Human epidermal growth factor receptor 2 (HER2/ERBB2), a critical member of the receptor tyrosine kinase (RTK) family, is altered in a subset of CCA, with reported frequencies varying widely by cohort and assay [18,19]. However, current HER2-targeted agents (e.g., trastuzumab) have limited clinical efficacy in CCA, with responses reported mainly in HER2-amplified tumors. This suggests that mechanisms other than genomic amplification may contribute to aberrant HER2 activation. The activity of RTKs depends not only on their expression levels but also on precise vesicular trafficking, including endocytosis, lysosomal degradation, and membrane recycling [20,21,22]. Under normal conditions, activated HER2 is internalized and transported to lysosomes for degradation, thereby terminating signal transduction [23,24,25]. HER2 trafficking is controlled by several regulators, including HSP90 chaperones [25], SORLA-dependent sorting, Rab11-positive recycling endosomes, and clathrin/AP-2-mediated internalization [20,21,22,26,27,28,29]. Disruption of this process leads to persistent retention of HER2 at the plasma membrane and abnormal activation of downstream signaling [30,31]. However, studies on the regulation of HER2 trafficking in CCA are still scarce.

Adaptor-related protein complex 1 subunit sigma 3 (AP1S3) is a core component of the AP-1 complex, primarily responsible for intracellular vesicle sorting and spatial trafficking of transmembrane receptors [26]. AP1S3 expression has been reported to be altered in some cancers [27], and its dysfunction is associated with the pustular psoriasis [32,33]. However, the role of AP1S3 in CCA and whether it mediates the link between inflammation and cancer have not been reported.

We found that IL-17A and TNF-α, two cytokines elevated in psoriasis, increase AP1S3 while activating NF-κB/RELA in CCA cells. This does not establish that psoriasis drives AP1S3 in patients, nor does it establish direct transcriptional regulation: the evidence consists of a database prediction, weak correlations (R = 0.18–0.29) and a non-specific inhibitor. High AP1S3 expression is associated with the SCEL+ squamous CCA program, which may reflect a general squamous/keratinization program rather than a psoriasis-specific subtype, and SCEL induction alone does not prove transdifferentiation. These findings support a candidate mechanism that requires RELA loss-of-function, ChIP-qPCR, and promoter-reporter and binding-site mutation experiments, as well as validation in psoriasis patients, psoriasis-bearing mouse models and CCA cases with psoriasis.

## 2. Materials and Methods

### 2.1. Mendelian Randomization (MR) and Multi-Omics Association Analysis

Two-sample MR analysis was performed as an exploratory analysis in accordance with the STROBE-MR guidelines [34]. Summary statistics for psoriasis vulgaris (GCST90018907) [35] and hepatic bile duct cancer/cholangiocarcinoma (GCST90018803) [36] were obtained from the GWAS Catalog. Both are trans-ancestry meta-analyses of BBJ, UKB and FinnGen (psoriasis: 5072 European and 206 East Asian cases; CCA: 832 European and 418 East Asian cases); ancestry-specific summary statistics were not available for all variants, so the meta-analyzed estimates were used and this is acknowledged as a limitation. The two datasets share controls (up to 99.4% of European controls), which can bias two-sample MR; we therefore repeated the analysis in an independent psoriasis GWAS GCST90019016 [36], which does not include UKB, FinnGen or BBJ. Cross-trait LDSC was used as an empirical check (intercept 0.003, SE 0.0043; genetic correlation 0.284, SE 0.248).

Instrumental variables (IVs) were selected at *p* < 5 × 10^−8^ and clumped for linkage disequilibrium (LD; r^2^ < 0.001) within 10,000 kb using the 1000 Genomes Phase 3 European reference panel (*n* = 503). The 10,000 kb window is the TwoSampleMR default and was pre-specified; 1000 kb and 250 kb windows were analyzed as sensitivity analyses. Palindromic SNPs with minor allele frequency >0.42 were excluded. The extended MHC region (chr6:25,000,000–34,000,000, GRCh37) was excluded a priori because of long-range LD and pervasive pleiotropy; a sensitivity analysis retained MHC variants. All retained SNPs had F > 10. We did not apply an unstructured PhenoScanner filter because the trait list was not pre-specified and conditioning on downstream traits can introduce collider bias. Instead, confounder domains were defined a priori (smoking, alcohol, body mass index/obesity, type 2 diabetes, inflammatory bowel disease, primary sclerosing cholangitis, hepatitis B/C, cirrhosis/NAFLD and autoimmune traits), and pleiotropy was assessed with the MR-Egger intercept and MR-PRESSO. A sensitivity analysis excluded the established pleiotropic IL12B and TYK2 loci. The full IV list is provided in Supplementary MR Table. The inverse variance weighted (IVW) method was used as the primary estimator, with a random-effects model when Cochran’s Q *p* < 0.05. Weighted median, MR-Egger and weighted mode were used as sensitivity estimators. Heterogeneity was assessed by Cochran’s Q; horizontal pleiotropy by the MR-Egger intercept and MR-PRESSO (300 Monte Carlo simulations); and directionality by Steiger filtering. Leave-one-out analysis was performed for the strict IVW estimate. Reverse MR used CCA as the exposure and psoriasis as the outcome; 19 IVs were retained at *p* < 1 × 10^−5^ and only one at *p* < 5 × 10^−8^. Post hoc power was calculated for OR 1.12, and the OR detectable with 80% power was derived from the observed standard error. Analyses were performed in R 4.3.3 (TwoSampleMR/MRPRESSO) [37,38] and independently reproduced in Python 3.12. Because the primary psoriasis→CCA analysis was a single pre-specified hypothesis, no multiplicity correction was applied to it. Secondary MR (553 genes) and mediation (three mediators) were exploratory; Benjamini–Hochberg FDR and Bonferroni thresholds were used where indicated. If a Bonferroni correction was applied across all 659 exploratory MR tests (26 immune traits, 76 metabolite/protein traits, 553 genes, 3 mediators and the primary test), the threshold would be 7.6 × 10^−5^; the original *p* = 0.037/0.041 would not survive, whereas the strict primary *p* = 0.981 would be unchanged. The multiplicity inventory is provided in Supplementary Table S10.

Two-step MR mediation was exploratory. Path a (psoriasis→mediator) used psoriasis IVs and path b (mediator→CCA) used mediator IVs; the indirect effect was a × b, with Sobel SE and a 95% CI. The proportion mediated was a × b/c, where c is the total effect; 95% CIs were obtained by Monte Carlo simulation conditional on the observed total effect. Because the total effect was not robust and mediator instruments were weak, these estimates were not interpreted causally. A total of 553 co-upregulated genes were screened from the psoriasis (GSE54456) [39] and CCA (TCGA-CHOL) transcriptome datasets, and their cis-eQTL data (from the eQTLGen Consortium) were used for an exploratory secondary MR analysis. The skin–tumor intersection and cis-eQTL analyses were prioritization steps, not formal mediation analyses. Mediators were selected if path a and path b each had a nominal *p* < 0.05 in the exploratory screen; three mediators met this criterion. This is a data-driven filter, not formal mediator validation. After Benjamini–Hochberg correction across the three mediators, all q values were 0.146.

### 2.2. Clinical Transcriptomics and Single-Cell RNA Sequencing (scRNA-seq) Data Analysis

The limma package in R was used for normalization and differential expression gene (DEG) screening of the GSE54456 and TCGA-CHOL datasets. Kaplan–Meier survival analysis and gene set enrichment analysis (GSEA) were performed in combination with clinical and sequencing data from the Fu-iCCA cohort previously constructed by Dong et al. [40]. Differentially expressed genes were defined as log2 fold-change > 1 and Benjamini–Hochberg FDR < 0.05 in both GSE54456 and TCGA-CHOL; 553 genes met both criteria.

Six public CCA single-cell transcriptome datasets (GSE138709 [41], GSE151530 [42], GSE125449 [43], GSE181878 [44], GSE189903 [45], GSE171899 [46]) were integrated. Strict quality control (removal of low-quality cells with mitochondrial gene expression ratio > 10% or detected gene number < 200) and normalization were performed using the Seurat package (v4.3.0), and batch effects were corrected by the Harmony algorithm. After dimensionality reduction by principal component analysis (PCA), the UMAP algorithm was used for nonlinear visualization and unsupervised cell subpopulation annotation. The slingshot algorithm was used to order malignant cells along a computational pseudotime trajectory. Major lineages were annotated using canonical markers: epithelial (EPCAM, KRT19, KRT8, KRT18), immune (PTPRC, CD3D, CD68), fibroblast (COL1A1, COL3A1, DCN) and endothelial (PECAM1, VWF). Malignant epithelial cells were separated from non-malignant epithelial cells using inferCNV. The SCEL+ subpopulation was defined by SCEL, KRT13, KRT16 and KRT6A expression. Annotation was marker-based and was not independently validated by pathology, IHC or an external single-cell cohort (Table S14).

### 2.3. Clinical Specimen Collection and Cell Culture

Fresh tumor tissues and paired adjacent normal tissues were collected from 7 patients with pathologically confirmed CCA at the Second Xiangya Hospital of Central South University (this study was approved by the hospital ethics committee, and informed consent was obtained from all patients). HuCCT1 was purchased from the Cell Bank of the Chinese Academy of Sciences (Shanghai, China); QBC939 and 293T were purchased from Guangzhou Jennio Biotech Co., Ltd. (Guangzhou, China). All cell lines were authenticated by STR profiling and confirmed to be mycoplasma-free. Cells were cultured in DMEM or RPMI-1640 medium supplemented with 10% fetal bovine serum (FBS) and 1% penicillin-streptomycin, and incubated in a humidified incubator at 37 °C with 5% CO_2_. Cells were stimulated with recombinant human IL-17A (100 ng/mL) and TNF-α (50 ng/mL) as a mechanistic inflammatory stimulus. The concentrations of IL-17A (100 ng/mL) and TNF-α (50 ng/mL) used in this study were selected based on previous reports demonstrating robust activation of NF-κB signaling in cholangiocarcinoma cells. Although these concentrations are higher than typical serum levels in psoriasis patients (low pg/mL to tens of pg/mL) by approximately 10^2^–10^4^-fold (Table S12), they are within the range commonly used in in vitro mechanistic studies to achieve sustained pathway activation. Moreover, local tissue concentrations in psoriatic skin or inflamed bile ducts may be substantially higher than serum levels due to paracrine secretion. Nevertheless, these concentrations are supraphysiologic and were not used to infer circulating levels; no single-cytokine or dose–response experiments were performed in this revision. IHC was performed on the same seven paired formalin-fixed paraffin-embedded sections. Staining was quantified with the H-score (intensity 0–3 × percentage of positive cells; range 0–300); because only seven paired cases were available, the H-score was analyzed descriptively and no independent cutpoint was derived. The Fu-iCCA cohort included 262 patients with RNA-seq and overall survival data; stage and other clinicopathological covariates were not available in the processed public file. TCGA-CHOL contained 35 tumor RNA-seq samples from 51 cases, of which 33 had expression and overall survival data and were used for the Cox re-analysis (Table S8). BAY 11-7082 (10 μM) was used as a non-specific NF-κB pathway inhibitor; no RELA knockdown, knockout or dominant-negative approach, ChIP-qPCR, promoter-reporter assay or binding-site mutation was performed. The de-identified dataset contained only the diagnosis and specimen type; age, sex, tumor location, histological subtype/differentiation, TNM stage, HBV/HCV status, cirrhosis and psoriasis history were not available (Table S18). The cohort is a single-center convenience sample; consecutive enrolment was not recorded, no a priori power calculation was performed, and the analysis is descriptive and unadjusted. HuCCT1 (intrahepatic CCA) and QBC939 (bile duct CCA) were selected as two established human CCA lines that are STR-authenticated, mycoplasma-free, lentivirally transducible and express detectable AP1S3 and HER2; selection was not based on HER2 amplification status. HER2 protein was detectable by Western blot in both lines, but expression was not quantified against a calibration standard, and HER2 amplification was not tested by FISH, qPCR or copy-number analysis (Table S19). 293T cells were used only for lentiviral packaging and Co-IP, not as a CCA model. No non-malignant cholangiocyte line (H69 or HIBEC), additional CCA line (RBE, HuH28 or TFK-1) or patient-derived organoid was used (Table S20).

### 2.4. Lentiviral Packaging, Plasmid Construction, and Site-Directed Mutagenesis

Lentiviral shRNA vectors targeting AP1S3 (sh-AP1S3) and overexpression plasmids (oeAP1S3) were constructed. To explore the protein–protein interaction interface, the EIPDLL motif (residues 930–935) in the HER2 kinase domain was mutated to EIPDAA using a site-directed mutagenesis kit to generate the HER2-mut plasmid. A constitutively active HER2 plasmid (CA-HER2) was also constructed for rescue experiments. All plasmids were constructed by GeneChem Co., Ltd. (Shanghai, China). When cell confluence reached 70%, Lipofectamine 3000 (Thermo Fisher Scientific, Waltham, MA, USA) was used for transient transfection or lentiviral infection, followed by puromycin selection to obtain stably expressing cell lines.

### 2.5. Cell Proliferation, Migration, and Invasion Assays

Proliferation assays: Cell growth curves were plotted using the CCK-8 assay (measuring absorbance at 450 nm). The EdU cell proliferation kit was used to calculate the EdU-positive rate after Apollo fluorescence labeling and DAPI nuclear staining. For colony formation assays, 500–600 cells were seeded per well, cultured continuously for 1–2 weeks, and stained with crystal violet, and colonies containing more than 50 cells were counted.

Migration and invasion assays: Two-dimensional migration ability was evaluated by the wound healing assay, recording the serum-free scratch healing rate at 0 h and specific time points. Transwell chambers with 8 μm pore size were used (the upper chamber of the invasion group was pre-coated with Matrigel matrix), and medium containing FBS was added to the lower chamber as a chemoattractant. The number of penetrated cells was counted under a microscope after crystal violet staining.

### 2.6. Gene Expression and Protein Half-Life Chase (CHX/CQ Chase) Assay

Total RNA was extracted using TRIzol reagent and reverse transcribed. Quantitative real-time PCR (RT-qPCR) was performed using the SYBR Green method with GAPDH as the internal reference, and relative expression levels were calculated using the 2^−ΔΔCt^ method. Primer sequences are listed in Supplementary Table S22. Total cellular protein was extracted, quantified by BCA assay, separated by SDS-PAGE, and electrotransferred to PVDF membranes. After blocking, membranes were incubated sequentially with specific primary antibodies (AP1S3, HER2, p-HER2, all purchased from Proteintech) and HRP-conjugated secondary antibodies, and visualized by ECL chemiluminescence with grayscale quantification. Uncropped membranes with molecular-weight markers are provided in the Original gel file; quantification uses all biological replicates.

To determine the protein half-life and degradation pathway of HER2, cells were treated with the protein synthesis inhibitor cycloheximide (CHX, 100 μg/mL) alone or in combination with the lysosomal inhibitor chloroquine (CQ, 50 μM). Cell lysates were collected at predetermined time points, and HER2 expression was detected by Western blotting.

### 2.7. Immunofluorescence and Live-Cell Cross-Linking Co-Immunoprecipitation (Cross-Linking Co-IP)

Immunofluorescence (IF): Cell slides or paraffin sections were fixed with paraformaldehyde, permeabilized with Triton X-100, and blocked with BSA. After incubation with primary antibodies against target proteins and fluorescence-labeled secondary antibodies, nuclei were stained with DAPI and protein spatial localization was observed under a laser confocal microscope.

Live-cell cross-linking Co-IP: To capture transient vesicle transport complexes, live cells were pretreated with the reversible chemical cross-linker DSP (0.5 mM, incubated at room temperature for 30 min). After cell lysis, specific primary antibody or isotype IgG control was added, and immune complexes were enriched using Protein A/G magnetic beads. After thorough washing and the addition of reducing agent DTT to reverse DSP cross-linking, interacting proteins were identified by Western blotting. DSP cross-linking can capture proximity as well as direct binding; no recombinant protein/peptide binding assay (SPR, ITC or pull-down) was performed.

### 2.8. Cell Membrane Receptor Quantification and Recycling Endocytosis Kinetics Assay

Live cell surface proteins were specifically labeled with the cell-impermeable biotin Sulfo-NHS-SS-Biotin at 4 °C. After whole-cell lysis, membrane protein fractions were specifically precipitated using streptavidin agarose, eluted, and quantified by Western blotting to determine the abundance of HER2 on the cell membrane.

For the recycling endocytosis kinetics assay, cells were incubated at 37 °C to trigger endocytosis after cell surface biotinylation. Subsequently, residual biotin on the cell membrane surface was cleaved by incubation with 100 mM MesNa at 4 °C for 30 min, and MesNa activity was quenched with 5 mg/mL iodoacetamide. Cells were then incubated again at 37 °C to allow endocytic vesicles to recycle back to the membrane, and biotin labels recycled to the membrane were stripped at time gradients. Cells were lysed, and intracellular reduction-resistant biotinylated proteins were precipitated. The recycling rate of HER2 back to the membrane was calculated by quantifying the signal attenuation rate.

### 2.9. Subcutaneous Xenograft Model in Nude Mice

The animal experimental protocol was approved by the Experimental Animal Ethics Committee of the Second Xiangya Hospital of Central South University. CCA stable cell lines in logarithmic growth phase were collected, resuspended in an equal volume mixture of PBS and Matrigel, and subcutaneously inoculated into 4-week-old female BALB/c nude mice (purchased from Hunan Boruixin Biotechnology Co., Ltd., Changsha, Hunan, China). After tumor formation, mice were grouped and intervened according to the experimental design (stable AP1S3 knockdown group and HER2 reconstitution rescue group). Tumor length and width were measured every 3 days using a vernier caliper, and tumor volume was calculated as V = 0.5 × length × width^2^. At the end of the experiment, mice were sacrificed and tumor tissues were completely dissected, weighed, and photographed. Paraffin sections were prepared for immunofluorescence analysis of HER2 and p-HER2. The xenograft experiment used HuCCT1 cells and three groups, shNC, shAP1S3 and shAP1S3 + Flag-HER2, with *n* = 5 mice per group (15 total). Mice were grouped after tumor formation; the randomization method and blinding of tumor measurement/analysis were not documented, and no a priori power calculation was performed. Tumor volume was measured every 3 days and analyzed by two-way repeated-measures ANOVA (time × group, with mouse as the subject) with Bonferroni correction; endpoint tumor weight was a secondary one-way ANOVA with Tukey’s test. Endpoint-only comparisons were not used as the primary test, consistent with Section 2.11. HER2 reconstitution used exogenous Flag-HER2 overexpression, not CRISPR knock-in or the endogenous promoter, and was not titrated to the endogenous HER2 level; it may therefore exceed physiological expression. The rescue shows compensation, not restoration of physiological HER2. The design and reporting are summarized in Supplementary Table S15.

Key reagents and antibodies are listed in Supplementary Table S22.

### 2.10. Structure-Guided Drug Optimization and All-Atom Molecular Dynamics (MD) Simulation

High-throughput virtual screening targeting the candidate AP1S3 binding pocket was performed based on the Drugclip platform [47]. The initial hit STK040969 was selected by DrugCLIP Score and Vina Score. Iterative structure optimization was performed on the binding pose of the initial hit within the AP1S3 pocket: (1) replacement of a hydrophobic phenyl moiety with a cyclopropyl group at the P2 position; (2) elimination of one hydrogen bond acceptor to lower topological polar surface area (TPSA); and (3) reduction of rotatable bonds from 13 to 9. The computational candidate STK040969-opt was obtained, and its physicochemical properties and ADMET properties were predicted with ADMETlab 3.0 [48]. This workflow is described in Supplementary Computational Note 1. The pocket was not validated by mutagenesis, and the compound was not synthesized or experimentally tested; the docking, MD and ADMET results are computational predictions only. Chemical structures/SMILES and predicted properties are provided in the ADMETlab 3.0 reports (Supplementary Computational Note S1). Atom-level optimization records, receptor preparation, docking box, forcefields, water/ions, thermostat/barostat, random seeds and replicate trajectories were not fully archived; the campaign is therefore not fully reproducible (Table S16). Numeric ADMET values are provided in Table S21.

All-atom MD simulations were performed using the GROMACS 2022 software package. The ligand was parameterized with the GAFF2 forcefield and charges were assigned by the RESP method, and the receptor was parameterized with the AMBER14SB forcefield. The system was placed in a TIP3P water model environment with 0.15 M NaCl, and energy minimization was performed by the steepest descent and conjugate gradient methods. A 100 ns production simulation was carried out under the NPT ensemble (310 K, 1 bar). After extracting trajectory files, indicators such as root mean square deviation (RMSD), root mean square fluctuation (RMSF), and intermolecular hydrogen bond network distribution were calculated to comprehensively evaluate the dynamic stability of the complexes.

### 2.11. Statistical Analysis

Clinical cohort and in vitro/in vivo experimental data were statistically analyzed and plotted using IBM SPSS Statistics 26.0 and GraphPad Prism 9.0 software. For comparison of means between two groups, paired two-tailed Student’s *t*-test was used for paired clinical tissue samples; independent two-tailed Student’s *t*-test was used for independent samples conforming to normal distribution and homogeneity of variance, otherwise the non-parametric Mann–Whitney *U* test was applied. For comparison among multiple groups or multiple time points, one-way analysis of variance (one-way ANOVA) followed by Tukey’s or Dunnett’s test for pairwise comparisons was used for one-way comparison among multiple groups; two-way analysis of variance (two-way ANOVA) combined with Bonferroni post hoc test was performed for bivariate experiments involving time and treatment factors. Survival analysis was performed using the Kaplan–Meier method, and survival differences were compared with the log-rank test. Pearson or Spearman correlation coefficients were used for correlation analysis. A *p* value <0.05 was considered statistically significant. Exact two-sided *p* values and effect sizes with 95% CIs are reported in Table S17 where available, and in the archived Prism source files for figure panels; asterisks in figures are used only for compactness and correspond to *, *p* < 0.05; **, *p* < 0.01; ***, *p* < 0.001. Unless explicitly stated otherwise, n refers to biological replicates (independent cell passages, independent wells or individual mice), not technical replicates; technical replicates are not counted as n. Western blots show one representative membrane from n biological replicates, and quantification uses biological replicates. Normality and variance assumptions were assessed with Shapiro–Wilk and Levene tests where applicable; paired or unpaired *t* tests, Mann–Whitney U tests, one-way/two-way ANOVA with Tukey or Bonferroni correction, Kaplan–Meier/log-rank and Cox proportional-hazards models were used as appropriate. The proportional-hazards assumption was checked for Cox models. Multiplicity was handled with Tukey/Dunnett/Bonferroni for planned comparisons and Benjamini–Hochberg FDR for omics and MR; no correction was applied to single pre-specified tests, and exploratory *p* values are labelled as such.

## 3. Results

### 3.1. Exploratory Mendelian Randomization Suggests a Possible Association Between Psoriasis and Cholangiocarcinoma and Identifies AP1S3 as a Candidate Gene

To explore whether systemic inflammation is genetically linked to CCA, we performed two-sample MR as a hypothesis-generating analysis. Under the pre-specified strict threshold (*p* < 5 × 10^−8^) and after excluding the extended MHC, eight IVs remained; the IVW estimate was OR 1.00 (95% CI 0.83–1.21; *p* = 0.98). When MHC variants were retained, 14 IVs gave OR 1.08 (95% CI 0.99–1.18; *p* = 0.11). The original *p* < 1 × 10^−5^ set (50 IVs) gave OR 1.13 (95% CI 1.01–1.26; *p* = 0.041), but this estimate was not robust in an independent psoriasis GWAS (49 IVs; OR 1.05, 95% CI 0.96–1.14; *p* = 0.30; MHC-included OR 1.07, 95% CI 0.99–1.15; *p* = 0.073). There was no evidence of heterogeneity or horizontal pleiotropy (Q *p* = 0.81; Egger intercept *p* = 0.54; MR-PRESSO *p* = 0.80), and all 14 strict IVs passed Steiger filtering. The strict analysis had only 21% power to detect OR 1.12, and 80% power only for OR ≥ 1.32. Reverse MR did not detect an effect of CCA on psoriasis (OR 1.01, 95% CI 0.98–1.05; *p* = 0.43), but only one IV remained at *p* < 5 × 10^−8^. Thus, the MR analysis is inconclusive and does not establish causality. This estimate would not survive Bonferroni correction across 659 exploratory tests (threshold 7.6 × 10^−5^; Supplementary Table S10).

Exploratory two-step MR mediation yielded mediated proportions of 10.8% for GPE (p-16:0/18:2; GCST90300318; 95% CI −2.4 to 66.6), 17.2% for CD127 on CD8+ T cells (GCST90001929; −6.3 to 108.6) and 16.8% for CD4−CD8− T cells (GCST90001613; −5.0 to 107.3); all Sobel *p* values were >0.09 and all indirect-effect CIs included zero. The directions were internally consistent (a < 0, b < 0, indirect effect > 0), but these mediators are not established causal intermediates, and CD4−CD8− T cells should not be uniformly described as anti-tumor. These analyses are reported in Supplementary MR Table S3 and Supplementary Figure S1C and are not used to support a causal chain. After Benjamini–Hochberg correction across the three mediators, all q values were 0.146.

**Figure 1:**
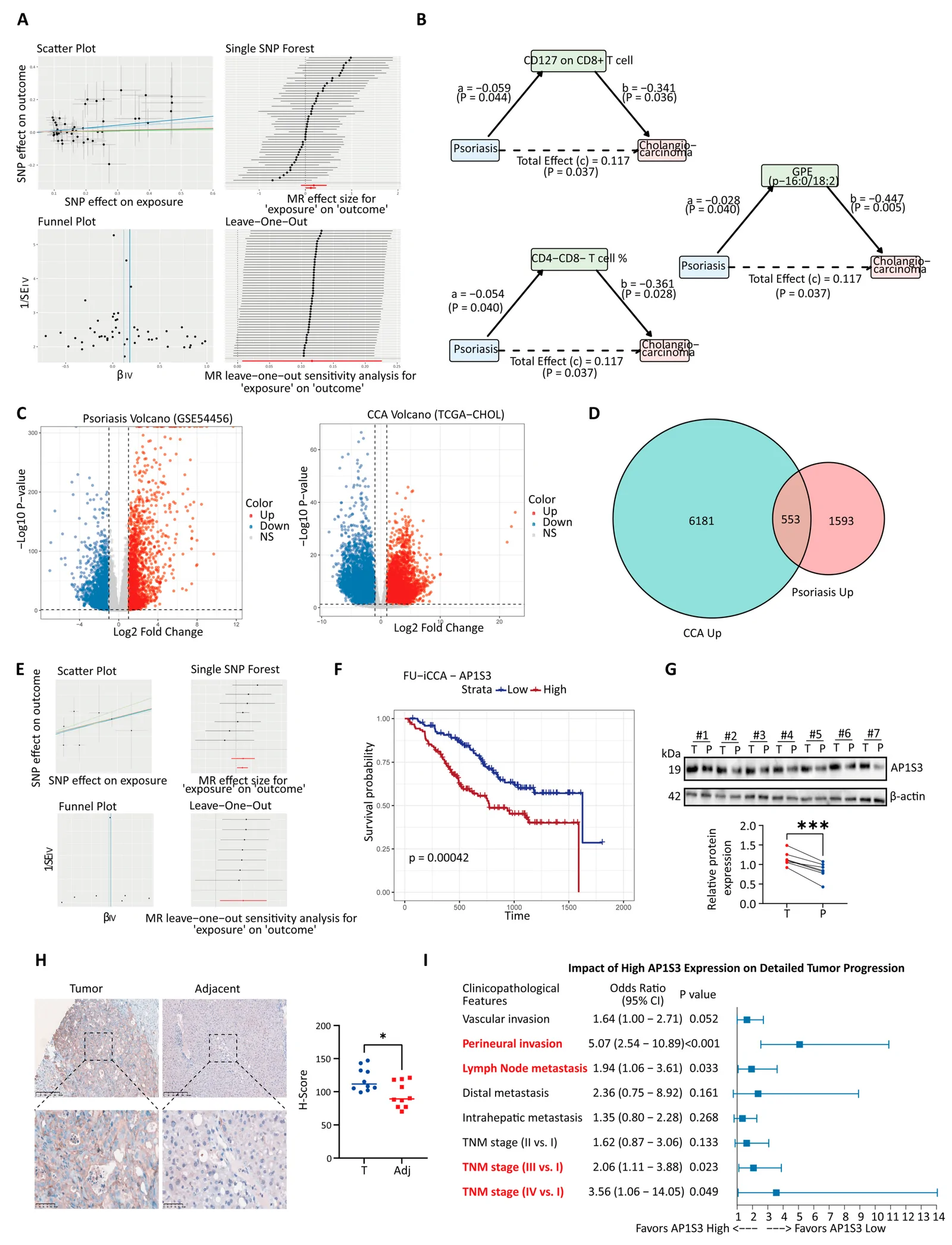
Exploratory Mendelian randomization and identification of AP1S3 as a candidate gene linking psoriasis to cholangiocarcinoma. (**A**) Two-sample Mendelian randomization (MR) analysis of the original *p* < 1 × 10^−5^ estimate (shown for transparency); the pre-specified strict estimate (*p* < 5 × 10^−8^, MHC excluded) and all sensitivity analyses are presented in Supplementary Figure S1A. (**B**) Exploratory two-step MR mediation analysis; proportions mediated are shown and 95% CIs are reported in Supplementary MR Table S3. (**C**) Volcano plots showing differentially expressed genes (DEGs) in psoriasis (GSE54456) and CCA (TCGA-CHOL) transcriptomes. (**D**) Venn diagram showing 553 co-upregulated DEGs between psoriasis and CCA. (**E**) Cis-eQTL-MR analysis examining the association between AP1S3 expression and CCA risk. (**F**) Kaplan–Meier survival analysis showing overall survival (OS) differences between patients with high and low AP1S3 expression in the Fu-iCCA cohort, analyzed by log-rank test. (**G**) Western blotting of AP1S3 in 7 paired CCA tumor and adjacent normal tissues from our center, with β-actin as the loading control; the blot is representative of the 7 paired patients (independent experimental replication is not possible for patient tissues). Clinical characteristics (age, sex, stage, histology, HBV/HCV, psoriasis history) were not available in the de-identified dataset; the analysis is descriptive and underpowered. (**H**) Immunohistochemical (IHC) detection of AP1S3 expression and localization in CCA and adjacent tissues (7 paired cases), with H-score quantification on the right; the small sample size and descriptive scoring are stated in the Limitations. Clinical characteristics were not available; H-score is descriptive and unadjusted (Table S18). (**I**) Forest plot of associations between high AP1S3 expression and clinicopathological features in the Fu-iCCA cohort (binary logistic regression). Perineural invasion, lymph node metastasis and advanced TNM stage were significant; vascular invasion was borderline (*p* = 0.052), and distant or intrahepatic metastasis were not significant. The direction label has been corrected: left favors lower AP1S3 and right favors higher AP1S3. * *p* < 0.05, *** *p* < 0.001.

To identify a candidate molecular link between psoriasis and CCA, we intersected differentially expressed genes from psoriasis lesions (GSE54456) and CCA tumors (TCGA-CHOL). Genes were defined as log2 fold-change > 1 and Benjamini–Hochberg FDR < 0.05 in both datasets, yielding 553 co-upregulated genes (Figure 1C,D). This intersection is a prioritization filter, not evidence of a shared mechanism. Secondary cis-eQTL MR of these genes used nominal *p* < 0.05 only for prioritization and identified nine genes (Supplementary Figure S2; Table S11). Survival analysis in the Fu-iCCA cohort then used *p* < 0.05 and HR > 1, retaining only AP1S3 (Supplementary Figure S3). This sequential 553→9→1 filter is data-driven and can inflate apparent effects. AP1S3 cis-eQTL MR (7 SNPs; F median 46.1, minimum 31.6) gave OR 1.53 (95% CI 1.07–2.20; *p* = 0.020), but the estimate was driven by one SNP (rs6436440; OR 1.58, 95% CI 1.03–2.42; *p* = 0.037) and did not survive Bonferroni correction for the nine candidates (*p* < 0.0056) or the 553-gene screen (*p* < 9.0 × 10^−5^). AP1S3 is therefore a candidate prioritized by convergent transcriptomic and survival evidence, not a validated mediator or an FDR-controlled causal gene. Analysis of the Fu-iCCA cohort demonstrated that high AP1S3 expression was significantly positively correlated with multiple adverse pathological features such as perineural invasion, lymph node metastasis, and advanced TNM stage (Figure 1I). These results suggest that AP1S3 is not only a prognostic biomarker for CCA but also may play an important role in its malignant progression.

### 3.2. AP1S3 Promotes the Malignant Phenotype of Cholangiocarcinoma Cells In Vitro

Adaptor-related protein complex 1 subunit sigma 3 (AP1S3) encodes the σ1C subunit of the AP-1 complex and is a core component regulating intracellular vesicle sorting and spatial trafficking of transmembrane receptors. An exploratory TCGA pan-cancer comparison (Supplementary Figure S4) showed higher AP1S3 expression in several tumor types, including CCA; this is descriptive and does not establish a conserved oncogenic role.

**Figure 2:**
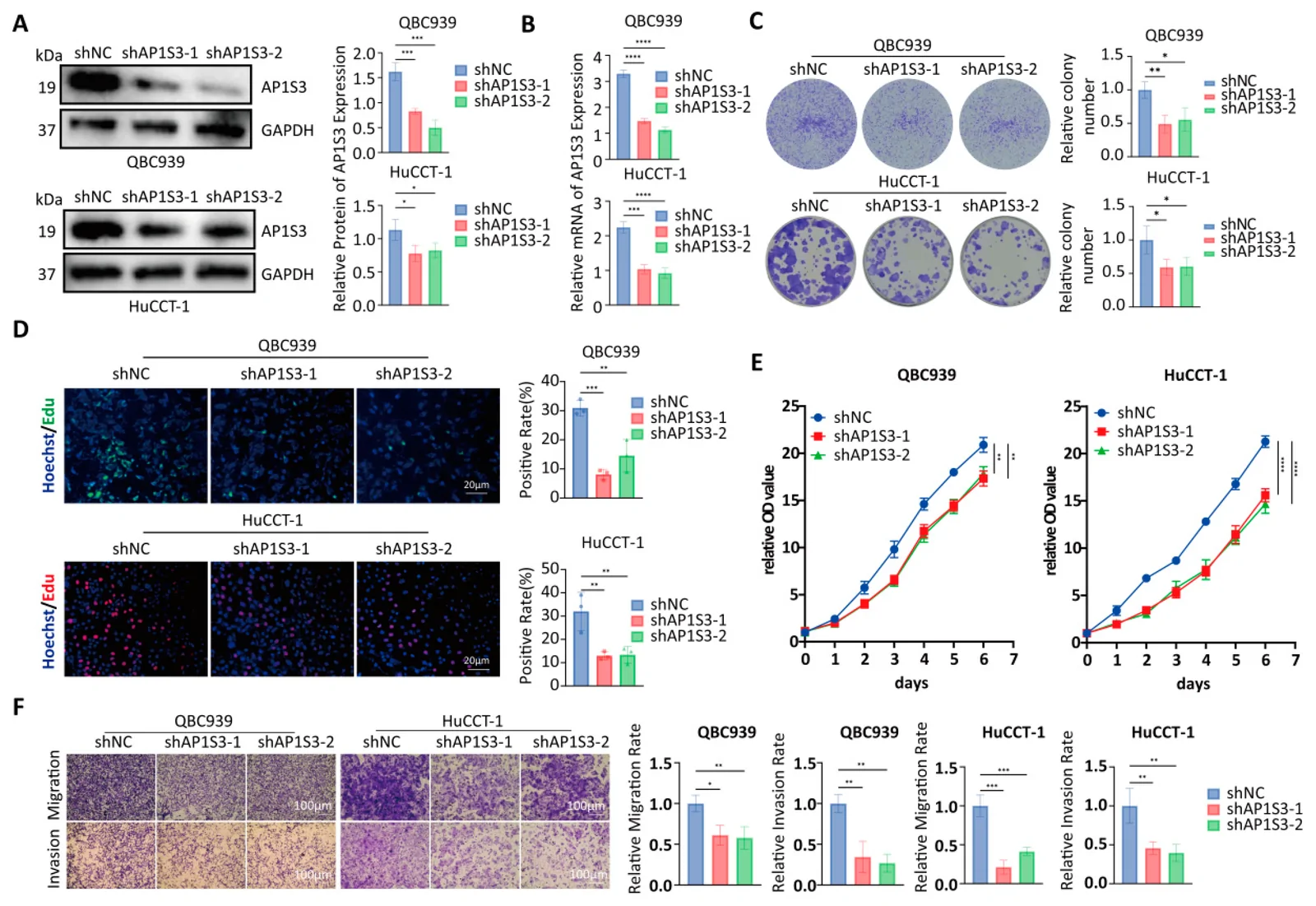
AP1S3 significantly promotes the proliferation, migration, and invasion of cholangiocarcinoma cells in vitro. (**A**,**B**) Western blotting (**A**) and qPCR (**B**) verifying the stable knockdown efficiency of AP1S3 in HuCCT1 and QBC939 cells (shNC as negative control, shAP1S3-1/2 as two independent shRNAs), with GAPDH as the loading control; data are presented as mean ± standard deviation of 3 independent experiments. (**C**) Colony formation assay detecting the effect of AP1S3 knockdown on CCA cell colony formation ability; colonies containing more than 50 cells were counted after crystal violet staining, *n* = 3 biological replicates. (**D**) EdU incorporation assay detecting the effect of AP1S3 knockdown on CCA cell proliferation ability; proliferating cells were labeled with Apollo fluorescence, and nuclei were stained with DAPI; the percentage of EdU-positive cells was calculated, *n* = 3 biological replicates. (**E**) CCK-8 assay detecting the dynamic effect of AP1S3 knockdown on CCA cell proliferation viability, measured continuously for 6 days, with *n* = 5 technical replicates with 3 independent biological replicates; technical wells were averaged before analysis and are not counted as *n*. (**F**) Transwell assay detecting the effect of AP1S3 knockdown on CCA cell migration and invasion abilities (the upper chamber of the invasion group was pre-coated with Matrigel); the number of penetrated cells was counted after crystal violet staining—*n* = 3 biological replicates. Group comparisons were performed using one-way ANOVA with Dunnett’s test for the two shAP1S3 groups versus the shared shNC control; * *p* < 0.05, ** *p* < 0.01, *** *p* < 0.001, **** *p* < 0.0001.

To clarify the biological function of AP1S3 in CCA, we constructed two stable AP1S3 knockdown CCA cell lines (shAP1S3-1, shAP1S3-2), and the knockdown efficiency was verified by Western blotting and qPCR (Figure 2A,B). In vitro functional experiments showed that knockdown of AP1S3 significantly inhibited the colony formation ability (Figure 2C) and proliferation rate (Figure 2D,E) of HuCCT1 and QBC939 cells. Transwell assays confirmed that AP1S3 knockdown simultaneously reduced the migration and invasion abilities of CCA cells (Figure 2F).

Conversely, stable overexpression of AP1S3 in HuCCT1 and QBC939 cells (Supplementary Figure S5A,B) significantly enhanced their colony formation, proliferation, migration, and invasion capabilities (Supplementary Figure S5C–F). The above bidirectional experimental results consistently indicate that AP1S3 is a key molecule driving the malignant phenotype of CCA cells, and its high expression confers stronger proliferative and invasive potential to tumor cells.

### 3.3. IL-17A/TNF-α Stimulation Upregulates AP1S3 and Is Associated with NF-κB/RELA Activation and a SCEL+ Squamous Program

**Figure 3:**
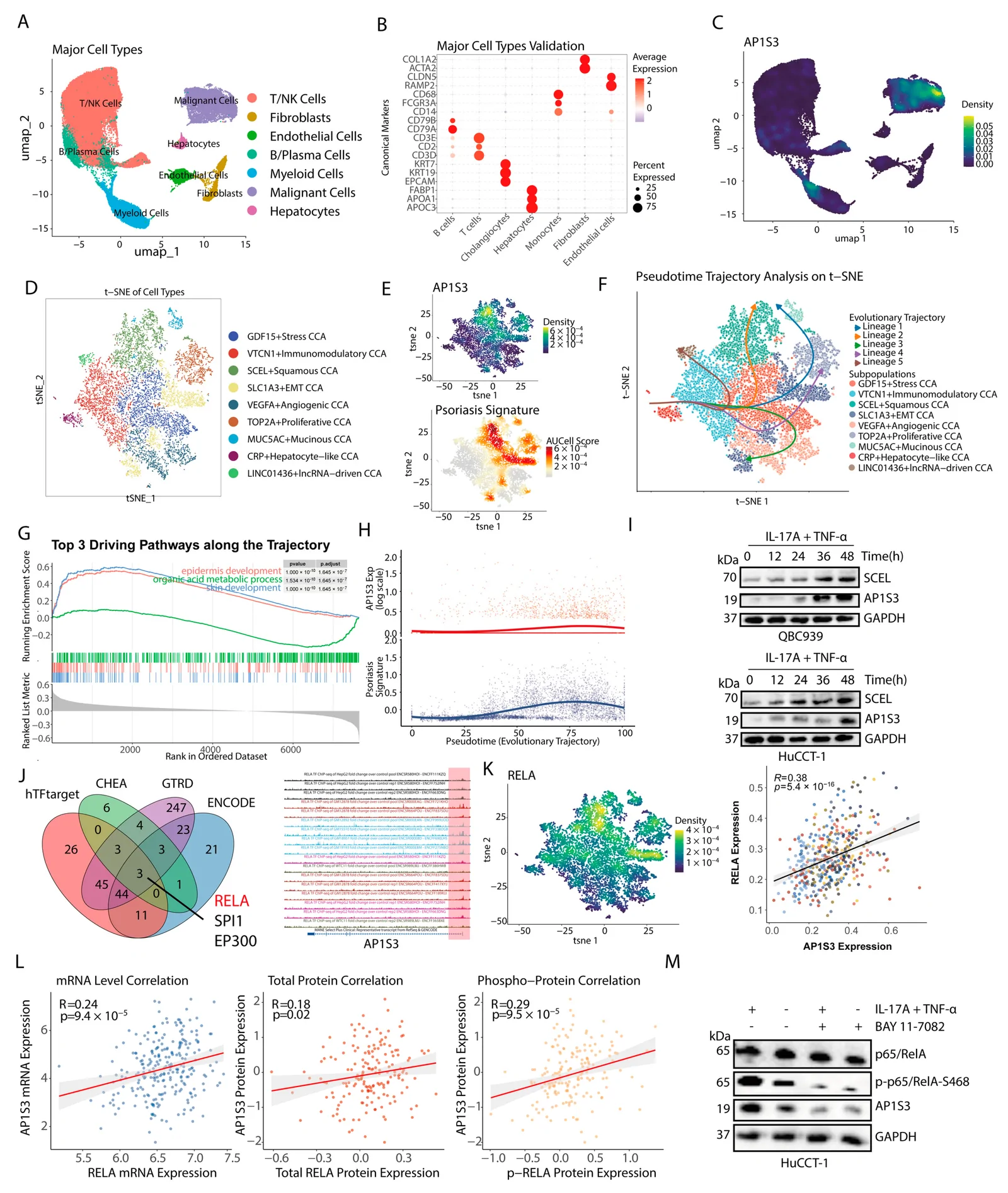
IL-17A/TNF-α stimulation upregulates AP1S3 and is associated with NF-κB/RELA activation and a SCEL+ squamous program in CCA. (**A**) Integration of 6 public CCA single-cell transcriptome datasets (GSE138709, etc.), followed by batch effect correction using the Harmony algorithm, UMAP dimensionality reduction clustering, and annotation of 7 major cell types. (**B**) Bubble plot verifying the expression of characteristic genes in each cell type. (**C**) UMAP plot showing the expression distribution of AP1S3 at the single-cell level. (**D**) Re-clustering and functional annotation of extracted malignant epithelial cell subpopulations, identifying 9 functionally heterogeneous CCA subpopulations. (**E**) UMAP plot showing the co-localization of AP1S3 and a psoriasis lesion-associated inflammatory/keratinization gene set (not disease-specific) in malignant subpopulations. (**F**) Pseudotime trajectory of malignant cells constructed by the slingshot yielding five computational trajectories; pseudotime is an inferred ordering, not evidence of differentiation. (**G**) GSEA showing the top 3 core pathways upregulated along pseudotime in trajectory 2. (**H**) Line plot showing the pseudotime expression changes in AP1S3 and inflammatory/keratinization gene set along trajectory 2. (**I**) Western blotting detecting the time-dependent changes in SCEL and AP1S3 protein expression in HuCCT1 and QBC939 cells after combined stimulation with IL-17A (100 ng/mL) + TNF-α (50 ng/mL); these supraphysiologic concentrations were used as a mechanistic stimulus, not to model circulating levels. (**J**) Computational prediction of upstream transcription factors of AP1S3 using GTRD/CHEA/hTFtarget/ENCODE, and UCSC track showing a RELA binding peak; this is hypothesis-generating and not experimental evidence of direct binding. (**K**) Single-cell metacell algorithm analyzing the expression correlation between RELA and AP1S3. (**L**) Pearson correlation analysis showing the correlations between RELA and AP1S3 at the mRNA, total protein, and phosphorylated protein (p-RELA-S468) levels in the Fu-iCCA cohort; correlations were weak (R = 0.18–0.29; R^2^ ≈ 0.03–0.08), indicating that RELA explains only a small fraction of AP1S3 variance. (**M**) Western blotting of AP1S3 after BAY 11-7082 (10 μM) pretreatment; BAY 11-7082 is not RELA-specific, and no RELA knockdown/knockout/dominant-negative, ChIP-qPCR or promoter-reporter assay was performed. Blots are representative of 3 independent experiments; quantification used all 3 biological replicates; and uncropped membranes with molecular-weight markers are provided in the Original gel file. *p* < 0.05.

Further clustering of malignant subpopulations showed that AP1S3 and a psoriasis lesion-associated inflammatory/keratinization gene set scored highest in the SCEL+ squamous cluster (Figure 3D,E). This gene set is not disease-specific: it overlaps with general inflammatory and keratinization programs, and the SCEL+ cluster is defined by squamous markers including KRT13, KRT16, KRT6A and SCEL. The co-localization therefore indicates that AP1S3 is expressed in a squamous-like CCA subpopulation with an inflammatory/keratinization program; it does not establish that psoriasis is the driver. SCEL has been reported as a squamous/keratinization-associated marker in some squamous carcinomas [49,50], and small series suggest that squamous/adenosquamous CCA may have a worse prognosis [51,52]; neither is specific to CCA. We therefore use this cluster as a hypothesis-generating model rather than as evidence of a psoriasis-specific subtype. The SCEL+ cluster was defined by SCEL, KRT13, KRT16 and KRT6A; it is a marker-defined cluster, not a validated histological subtype.

Pseudotime trajectory analysis of malignant cells using Slingshot yielded five computational trajectories (Figure 3F). Trajectory 2 showed a correlation with AP1S3 and the inflammatory/keratinization gene set; this is a computational ordering of cells along an inferred path, not evidence of differentiation, lineage commitment or a psoriasis-specific program. GSEA confirmed that patients with high AP1S3 expression were significantly enriched in epidermal development-related pathways and downregulated in organic acid metabolism pathways (Figure 3G), consistent with a general squamous/keratinization program. Along the pseudotime ordering of trajectory 2, the expression of AP1S3 and inflammatory/keratinization gene sets gradually increased (Figure 3H); this is an association, not a causal trajectory.

In vitro experiments showed that combined stimulation with IL-17A and TNF-α upregulated the expression of SCEL and AP1S3 in CCA cells in a time-dependent manner (Figure 3I). These experiments show that the combination can induce AP1S3 and SCEL in CCA cells; they do not establish that either cytokine alone is sufficient, that the effect is NF-κB-specific, or that the concentrations are physiological, and SCEL induction alone does not establish squamous transdifferentiation. Combined prediction of multiple transcription factor databases [53] and UCSC database analysis suggested that the NF-κB subunit RELA (p65) could bind to the AP1S3 promoter (Figure 3J), but this is computational prediction, not experimental evidence of direct binding. Single-cell metacell analysis and Fu-iCCA cohort validation showed weak positive correlations between RELA and AP1S3 at the mRNA, total protein, and phosphorylated protein levels (R = 0.18–0.29; R^2^ ≈ 0.03–0.08; Figure 3K,L), indicating that RELA explains only a small fraction of AP1S3 variance. Pretreatment with BAY 11-7082 reduced cytokine-induced AP1S3 upregulation (Figure 3M), but BAY 11-7082 is not RELA-specific; no RELA knockdown, knockout or dominant-negative approach, ChIP-qPCR, promoter-reporter assay or binding-site mutation was performed. Direct transcriptional regulation by RELA is therefore not established. KRT5, KRT14 and p63 were not tested, so the SCEL+ phenotype is not proven to represent squamous transdifferentiation. IL17RA and IL17RC transcripts were detectable in TCGA-CHOL tumors (median log2 counts 10.95 and 9.59), but receptor protein levels in HuCCT1 and QBC939 were not measured.

### 3.4. AP1S3 Interacts with HER2 Through the EIPDLL Motif and Modulates Its Lysosomal Degradation and Recycling

**Figure 4:**
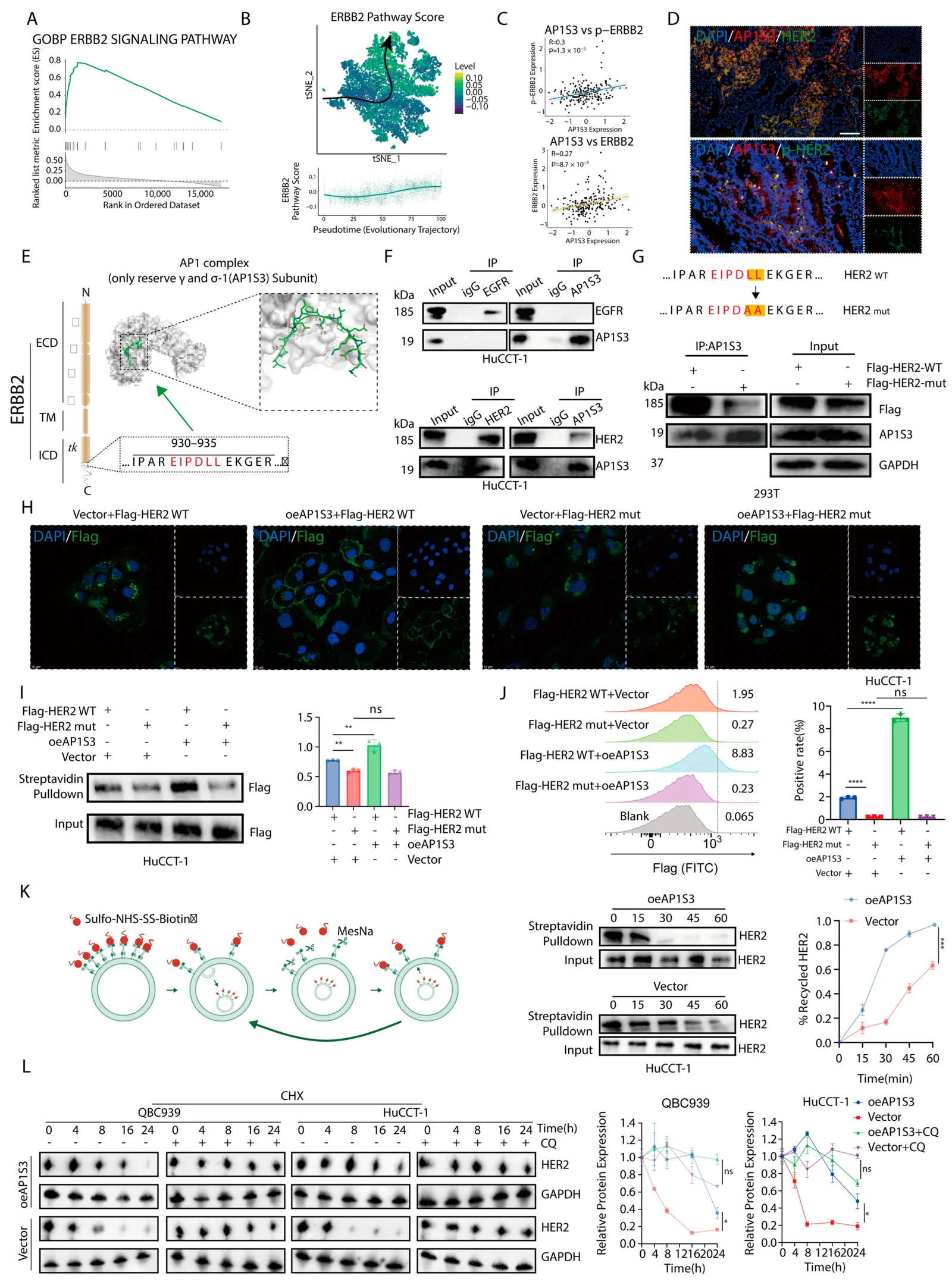
AP1S3 interacts with HER2 via the EIPDLL motif and modulates its lysosomal degradation and recycling. (**A**) GSEA showing significant enrichment of the ERBB2 signaling pathway in the high-AP1S3-expression group in the Fu-iCCA cohort. (**B**) Slingshot algorithm showing the trend of ERBB2 pathway score along pseudotime in trajectory 2 of malignant cells. (**C**) Pearson correlation analysis showing the protein expression correlations between AP1S3 and HER2, and between AP1S3 and p-HER2 in the Fu-iCCA cohort. (**D**) Immunofluorescence (IF) colocalization analysis of AP1S3 (green) and HER2/p-HER2 (red) in CCA tissues, with nuclei stained with DAPI (blue); scale bar = 20 μm. (**E**) The EIPDLL motif is at HER2 residues 930–935 within the kinase domain, not the C-terminal IDR (the original 1001–1015 annotation is incorrect); molecular docking visualizes a computational binding mode, not experimental binding; per-residue SASA is shown in Supplementary Figure S6; (**F**) Live-cell DSP cross-linking Co-IP assay detecting co-precipitation of AP1S3 with HER2/EGFR; DSP can capture proximity, so this is not a direct binding assay. (**G**) DSP cross-linking Co-IP after EIPDLL→EIPDAA mutation; the mutant may affect HER2 folding/phosphorylation, and surface level/degradation of the mutant without AP1S3 was not compared with wild-type. (**H**) IF detection of HER2 (red) subcellular localization in the control group, AP1S3 overexpression group, HER2-mut group, and AP1S3 overexpression + HER2-mut group; scale bar = 20 μm; representative images, not quantified; acquisition settings were kept constant within each experiment and brightness/contrast were adjusted uniformly across groups. (**I**) Cell surface biotinylation combined with streptavidin pulldown assay detecting the abundance of HER2 in the cell membrane fraction. (**J**) Flow cytometry detecting the proportion of HER2-high subpopulations in each group. (**K**) Biotin-based surface recovery assay measuring recovery of biotinylated HER2 after MesNa stripping; internalization controls, MesNa cleavage-efficiency controls and normalization to total surface HER2 were not included, so this is not a definitive recycling rate. (**L**) Cycloheximide (CHX, 100 μg/mL) protein half-life chase assay detecting the effect of AP1S3 overexpression on HER2 degradation, combined with lysosomal inhibitor chloroquine (CQ, 50 μM) to verify the degradation pathway. Both HuCCT1 and QBC939 expressed detectable HER2 protein; HER2 amplification was not determined (Table S19). Blots are representative of 3 independent experiments and quantification used all 3 biological replicates; uncropped membranes with molecular-weight markers are provided in the Original gel file. *p* < 0.05, * *p* < 0.01, ** *p* < 0.01; *** *p* < 0.001; **** *p* < 0.0001; ns, not significant.

To elucidate the molecular mechanism by which AP1S3 promotes CCA progression, we performed GSEA on the Fu-iCCA cohort and found that the high AP1S3 expression group was significantly enriched in the ERBB2 signaling pathway (Figure 4A), and the ERBB2 pathway score increased synchronously along the pseudotime progression of trajectory 2 (Figure 4B). Correlation analysis confirmed that AP1S3 expression was significantly positively correlated with the expression of HER2 and phosphorylated HER2 (p-HER2) (Figure 4C), which was further supported by tissue immunofluorescence colocalization results (Figure 4D).

As a vesicle-sorting protein, AP1S3 regulates the trafficking of transmembrane receptors by recognizing [D/E]XXXL[L/I/M]motifs [54,55,56]. Sequence alignment showed that the EIPDLL motif is present in HER2 at residues 930–935, within the kinase domain, not in the C-terminal intrinsically disordered region (IDR); EGFR lacks this exact motif. Molecular docking suggested a possible binding mode (Figure 4E), but docking is computational and does not demonstrate binding. DSP cross-linking Co-IP showed that AP1S3 co-precipitated with HER2 but not EGFR (Figure 4F); because DSP can capture proximity, this is not proof of direct binding. The mutation of EIPDLL to EIPDAA reduced co-IP (Figure 4G), but the mutant may alter HER2 folding, phosphorylation or other interactions, and its surface level and degradation without AP1S3 were not compared with wild-type HER2. The EIPDLL motif is partially buried in the HER2 kinase structure (I931 and L935 SASA ≈1 Å^2^; Figure 4E and Supplementary Figure S6), and its accessibility was not tested experimentally. AP1S3 is one of three σ1 isoforms (AP1S1, AP1S2, AP1S3; 69–87% sequence identity); AP1S1 is the most highly expressed isoform in TCGA-CHOL (median log2 11.35 vs 8.58 for AP1S3), so specificity for AP1S3 over AP1S1/AP1S2 is not established. The requirement for AP-1 γ/β subunits was not tested. AP1S3 is the σ1C subunit of the heterotetrameric AP-1 complex (γ, β1, μ1 and σ1); the current experiments cannot distinguish an AP1S3-specific effect from altered AP-1 assembly or function. AP1S1/AP1S2 and AP-1 γ/β were not depleted, and AP-1 complex integrity was not assessed.

Immunofluorescence detection showed that overexpression of AP1S3 significantly enhanced the membrane localization of HER2, while the membrane localization of mutant HER2-mut was no longer regulated by AP1S3 (Figure 4H). Cell surface biotinylation combined with streptavidin pulldown assay (Figure 4I) and flow cytometry (Figure 4J) further confirmed that the abundance of HER2 protein in the cell membrane fraction and the proportion of HER2-high cell subpopulations were significantly increased in the AP1S3 overexpression group.

**Figure 5:**
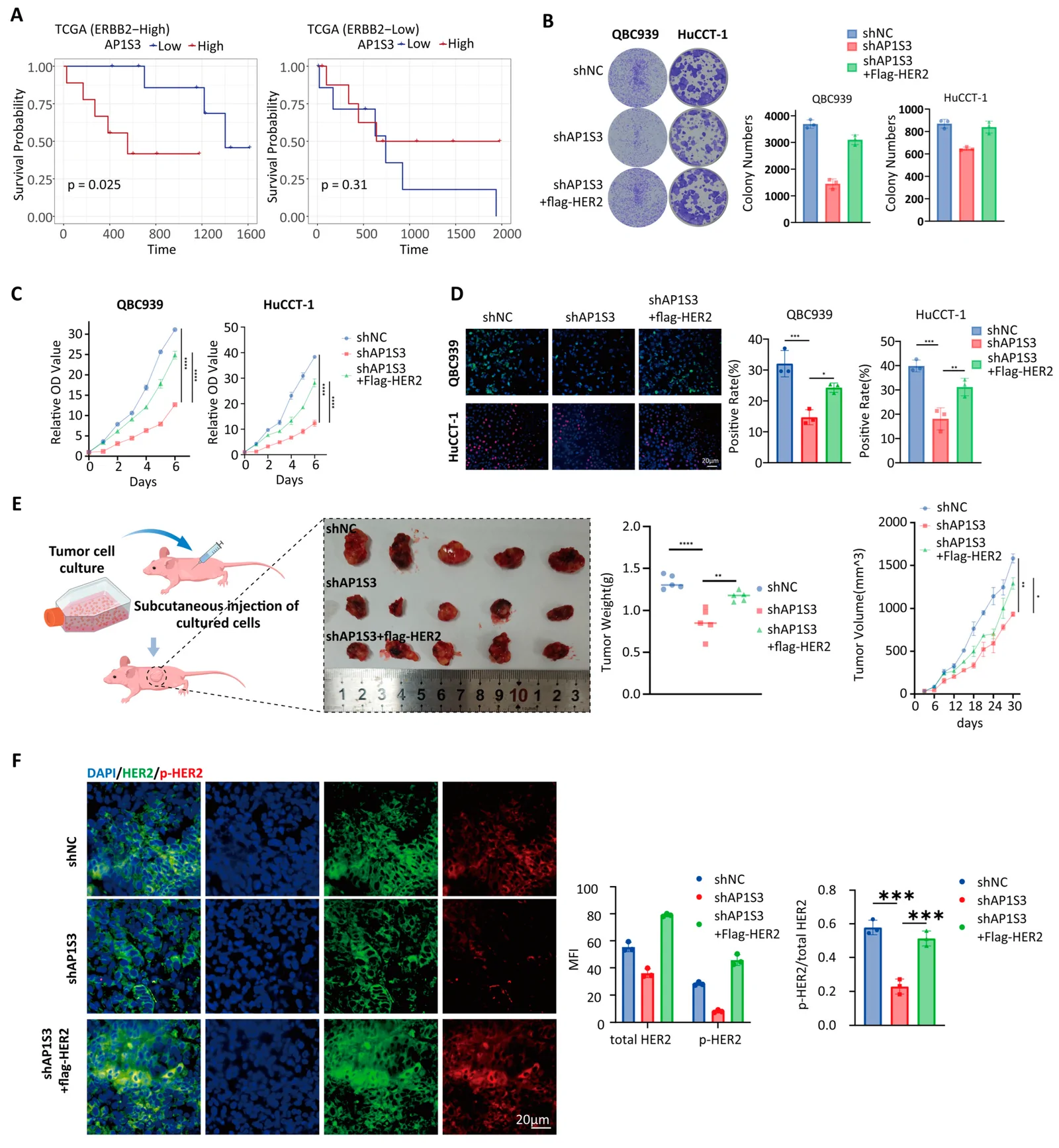
TCGA-CHOL reanalysis and HER2 rescue of the AP1S3 knockdown phenotype. (**A**) Re-analysis of TCGA-CHOL (33 tumors with expression and overall survival; 18 deaths). Kaplan–Meier curves for AP1S3-high vs. AP1S3-low in ERBB2-high and ERBB2-low subgroups; neither subgroup log-rank test was significant (*p* = 0.12 and *p* = 0.34), and the AP1S3 × ERBB2 interaction was *p* = 0.066 (Wald)/*p* = 0.029 (likelihood ratio) unadjusted and *p* = 0.314/*p* = 0.230 after stage adjustment (Table S8). (**B**) Colony formation assay detecting the colony formation ability of CCA cells after AP1S3 knockdown and Flag-HER2 reconstitution, *n* = 3 biological replicates. (**C**) CCK-8 assay detecting the effect of AP1S3 knockdown and Flag-HER2 reconstitution on CCA cell proliferation viability, with five technical wells per experiment with three independent biological replicates; technical wells were averaged before analysis and not counted as *n*. (**D**) EdU assay detecting the effect of AP1S3 knockdown and Flag-HER2 reconstitution on CCA cell proliferation ability, *n* = 3 biological replicates. (**E**) Subcutaneous xenograft model in nude mice: HuCCT1 cells from the shNC, shAP1S3 and shAP1S3 + Flag-HER2 groups were injected subcutaneously (*n* = 5 per group; 15 mice total). Tumor volume was measured every 3 days (V = 0.5 × length × width^2^), and tumors were weighed at the end of the experiment. Mice were grouped after tumor formation; randomization and blinding were not documented. Tumor volume was analyzed by two-way repeated-measures ANOVA (time × group, with mouse as the subject) with Bonferroni correction; endpoint tumor weight was a secondary one-way ANOVA with Tukey’s test; endpoint-only comparisons were not used as the primary test. HER2 reconstitution used exogenous Flag-HER2 overexpression, not knock-in and not titrated to endogenous HER2; the rescue therefore indicates compensation, not restoration of physiological expression. (**F**) IF detection of HER2 (green) and p-HER2 (red) expression in nude mouse xenograft tissues, with nuclei stained with DAPI (blue); scale bar = 20 μm, quantified as integrated density per field normalized to DAPI (*n* = 5 tumors/group), with quantitative analysis on the right. Group comparisons were performed using one-way ANOVA combined with Tukey’s test, * *p* < 0.05, ** *p* < 0.01, *** *p* < 0.001, **** *p* < 0.0001.

The biotin-based surface recovery assay showed increased recovery of HER2 at the membrane after AP1S3 overexpression (Figure 4K), but without internalization controls, MesNa cleavage-efficiency controls and normalization to total surface HER2, this cannot be interpreted as a true recycling rate. Cycloheximide (CHX) chase experiments showed that the overexpression of AP1S3 prolonged HER2 half-life, and the difference disappeared after chloroquine (CQ) treatment (Figure 4L). However, CQ is not specific: it blocks lysosomal acidification and also affects endosomal trafficking and autophagy, and it cannot distinguish lysosomal from autophagic degradation. No bafilomycin A1 or MG132 control was included. These results support a model in which AP1S3 overexpression modulates HER2 trafficking; they do not establish direct binding, AP1S3 specificity, active promotion of recycling, or whether EIPDLL is the sole interface. The reverse experiment (AP1S3 knockdown → HER2 surface level/recycling) was not performed; Figure 5 tests proliferation and HER2 rescue, not trafficking. No LAMP1, Rab4, Rab11 or EEA1 colocalization and no HER2 ubiquitination analysis were performed. The EIPDAA mutant may affect HER2 folding or phosphorylation; no control mutation outside the motif and no wild-type versus mutant comparison in an AP1S3-null background were performed.

### 3.5. HER2 Overexpression Rescues the Proliferation Defect of AP1S3 Knockdown In Vitro and In Vivo

To explore whether the AP1S3 effect might depend on HER2, we re-analyzed TCGA-CHOL. In the public dataset with 33 tumors and 18 deaths, AP1S3-high was not significantly associated with overall survival in either the ERBB2-high (log-rank *p* = 0.12) or ERBB2-low (*p* = 0.34) subgroup. The AP1S3 × ERBB2 interaction was *p* = 0.066 according to the Wald test and *p* = 0.029 with the likelihood-ratio test without adjustment, *p* = 0.074/*p* = 0.031 after adjustment for age and sex, and *p* = 0.314/*p* = 0.230 after adjustment for stage (23 cases, 8 deaths; Table S8). The original subgroup result shown in Figure 5A was therefore not reproducible; the analysis is underpowered and should be considered hypothesis-generating.

In vitro, the decreased colony formation and reduced proliferation of CCA cells caused by AP1S3 knockdown were reversed by exogenous HER2 overexpression. However, rescue by HER2 overexpression shows only that additional HER2 can compensate for AP1S3 loss; it does not prove that AP1S3 acts through HER2. The key experiments were not performed: HER2 depletion in AP1S3-knockdown cells, AP1S3 overexpression in HER2-null or HER2-mutant cells, and HER2 inhibitor (tucatinib, lapatinib or trastuzumab) treatment. Migration and invasion rescue were not tested. The HER2 amplification/expression status of HuCCT1 and QBC939 was not determined. Downstream p-AKT, p-ERK and p-HER3 were not measured, and the phospho-HER2 antibody/residue is not specified; the ‘sustaining oncogenic signaling’ claim therefore rests on p-HER2 alone. The xenograft used *n* = 5 per group, one cell line, no blinding or randomization details, and no orthotopic or immunocompetent model. These data support a candidate HER2-linked proliferation model, not a demonstrated HER2-dependent mechanism. The HER2 rescue used exogenous Flag-HER2 overexpression rather than knock-in or endogenous-promoter reconstitution, and expression was not titrated to the endogenous level; it may therefore exceed physiological HER2. The rescue demonstrates compensation, not restoration of physiological expression. Randomization and blinding were not documented, and no a priori power calculation was performed.

## 4. Discussion

This study proposes a candidate mechanism by which systemic inflammatory signaling may contribute to CCA progression. By integrating multi-omics data with rigorous biological validation, we identified an association between IL-17A/TNF-α signaling, AP1S3 and HER2 trafficking; the mechanism remains candidate-level and requires direct binding, loss-of-function and clinical validation. The computational section is a supplementary proof-of-concept, not a validated therapeutic strategy.

Using large-scale MR, we explored whether genetic liability to psoriasis is associated with CCA. The original relaxed-threshold estimate was modest and fragile; under strict instrument selection and MHC exclusion, the estimate was null (OR 1.00), and it was not replicated in an independent exposure dataset (OR 1.05). The study had limited power (21% for OR 1.12), so the MR analysis should be regarded as inconclusive and hypothesis-generating rather than as evidence of a causal effect. The association is biologically plausible because psoriasis is characterized by systemic IL-17A and TNF-α signaling, but larger CCA GWAS, ancestry-specific analyses and HLA fine-mapping will be required to resolve this question. We therefore focus the mechanistic work on AP1S3 as a candidate molecular link between psoriasis-associated inflammation and CCA, independent of the MR estimate.

Several features of the MR-to-AP1S3 link limit its interpretation. The intersection analysis compared psoriasis skin-lesion transcriptomes (GSE54456) with TCGA-CHOL tumor tissue; these are different tissues, platforms and disease contexts, and co-upregulation does not establish a shared mechanism. The cis-eQTL MR used eQTLGen blood expression, which may not reflect biliary epithelium or tumor tissue, and it rested on only seven SNPs. We did not perform colocalization because full regional eQTL data from biliary tissue were not available; LD confounding therefore cannot be excluded. The sequential selection of 9 genes from 553, followed by a survival filter, is data-driven and can inflate apparent effects, even though the survival analysis used an independent Fu-iCCA cohort. We therefore do not claim that AP1S3 formally mediates the effect of psoriasis on CCA; it is a candidate prioritized for functional study.

We found that IL-17A and TNF-α, two cytokines elevated in psoriasis, increase AP1S3 while activating NF-κB/RELA in CCA cells; the evidence is indirect. This does not establish that psoriasis drives AP1S3 in patients. High AP1S3 expression is associated with the SCEL+ squamous CCA program, which may reflect a general squamous/keratinization program rather than a psoriasis-specific subtype. These findings support a candidate mechanism that requires validation in psoriasis patients, psoriasis-bearing mouse models and CCA cases with psoriasis.

AP1S3 is a core subunit of the AP-1 complex, which is primarily recognized for its role in clathrin-mediated transport between the trans-Golgi network and endosomes. Our data suggest that AP1S3 modulates HER2 trafficking, but they do not establish active promotion of recycling. The CQ experiment is consistent with a lysosomal route, but CQ also affects endosomal trafficking and autophagy, and it cannot distinguish lysosomal from autophagic degradation. The surface recovery assay measures recovery of biotinylated HER2, not a true recycling rate, because internalization, MesNa efficiency and total surface HER2 were not controlled. We therefore cannot distinguish active promotion of recycling from passive protection from degradation. We do not propose that AP1S3 directly catalyzes recycling vesicle fusion; a plausible model is that it diverts HER2 away from a degradative fate, allowing the default recycling machinery (e.g., Rab4/Rab11) to operate on a larger substrate pool.

The core innovation of this study lies in proposing a model of HER2 regulation through post-translational trafficking. We found that AP1S3 overexpression is associated with reduced lysosomal degradation and increased surface HER2, through an interaction that requires the EIPDLL motif (residues 930–935, kinase domain). Direct binding, motif accessibility and AP1S3 specificity over AP1S1/AP1S2 remain to be established; the data support a trafficking model rather than a structurally defined interface or an active recycling mechanism. This model may help explain HER2 activation beyond gene amplification, but it requires recombinant binding, loss-of-function, live-cell imaging, ubiquitination and surface-residency experiments before the interface can be considered validated.

A computational proof-of-concept for targeting the candidate AP1S3–HER2 interface is presented in Supplementary Computational Note S1 and Supplementary Figure S7. It is based on docking, a single 100 ns MD simulation per complex and ADMET predictions; it has no experimental validation. The docking-score difference is within Vina error, the MD analysis has no replicates or free-energy calculation, and the compound has not been synthesized, tested by SPR or tested in cells. It should therefore be regarded as a computational hypothesis, not a lead compound or a promising therapeutic strategy. Because AP-1 is a shared adaptor, a small molecule targeting this site may also have broad off-target trafficking effects; this must be addressed experimentally.

Several limitations of this study should be acknowledged when interpreting the results. First, the immunodeficient nude mouse subcutaneous xenograft model used cannot fully replicate the complex systemic immune microenvironment of psoriasis patients. Future studies will employ immunocompetent spontaneous inflammation-driven CCA models for validation. Second, although pharmacological inhibition and bioinformatics analyses are consistent with RELA regulating AP1S3, the evidence is indirect and ChIP-qPCR, promoter-reporter and RELA loss-of-function experiments are still needed. Third, live-cell imaging of HER2-containing vesicles was not performed in this study; therefore, we cannot definitively distinguish whether AP1S3 actively promotes recycling or passively prevents lysosomal degradation. Future studies using HER2-GFP and endosomal markers (Rab11, LAMP1) are required to resolve this. Fourth, the concentrations of IL-17A (100 ng/mL) and TNF-α (50 ng/mL) used in our in vitro experiments are supraphysiological relative to serum levels, although they are standard in mechanistic studies in this field. Future experiments using lower, more physiologically relevant concentrations (e.g., 10–20 ng/mL) or conditioned media from psoriatic immune cells would strengthen the translational relevance. Fifth, we did not assess the effect of AP1S3 on HER2 ubiquitination, a key signal for lysosomal sorting (particularly K63-linked polyubiquitination). Future studies should directly measure HER2 ubiquitination levels under AP1S3 manipulation. Sixth, STK040969-opt is currently a computationally optimized proof-of-concept molecule; its druggability needs to be further verified through chemical synthesis, in vitro ADME assays, hERG patch-clamp testing, and in vivo pharmacokinetic studies. Seventh, the MR analysis was exploratory and underpowered: the strict estimate was null, the independent replication was not significant, the two Sakaue datasets shared controls, and HLA imputation was not performed. These limitations mean that the genetic epidemiological component should be interpreted as hypothesis-generating. Eighth, the AP1S3 cis-eQTL MR was nominal (*p* = 0.020) and did not survive correction for the 553-gene screen; it is therefore a prioritization signal rather than definitive causal evidence. Ninth, the skin–tumor intersection, blood-derived eQTLs, seven-SNP cis-eQTL MR, absence of colocalization and sequential 9/553 selection mean that AP1S3 is a candidate, not a formally validated mediator. Tenth, the clinical validation is limited: only seven paired tumors were available for WB/IHC, and CCA subtype (intrahepatic, perihilar or distal) was not recorded. Eleventh, no psoriasis patients, psoriasis-bearing mouse models or CCA cases with psoriasis were examined. Twelfth, the trafficking mechanism is not directly demonstrated. The study therefore cannot define the HER2 status of the models.

## 5. Conclusions

In summary, this study identifies a possible association between psoriasis and CCA and proposes a candidate cross-organ mechanism: inflammatory cytokines increase AP1S3, and AP1S3 is associated with reduced HER2 lysosomal degradation and increased surface HER2 through the EIPDLL motif. Direct binding, active recycling and AP1S3 specificity remain to be established. The candidate AP1S3–HER2 interface provides a framework for future experimental therapeutic development; the computational compound requires synthesis, binding and cellular validation. The MR association is exploratory and requires validation in larger, non-overlapping cohorts.

## Data Availability

Publicly available datasets used in this study include the following: The Cancer Genome Atlas cholangiocarcinoma dataset (TCGA-CHOL; accessed via GDC portal, https://portal.gdc.cancer.gov/ (accessed on 1 November 2026)); Gene Expression Omnibus (GEO) datasets GSE54456 (psoriasis transcriptome), GSE138709, GSE151530, GSE125449, GSE181878, GSE189903, and GSE171899 (single-cell RNA-seq of cholangiocarcinoma); GWAS summary statistics for psoriasis (GCST90018907 and GCST90019016) and cholangiocarcinoma (GCST90018803) and GPE (GCST90300318) from the GWAS Catalog (https://www.ebi.ac.uk/gwas/ (accessed on 1 November 2026)); mediation analysis datasets from GCST90027446–GCST90027857, ebi-SMB150-GCST90133164–GCST90133313, GCST90199621–GCST90201020, ieu-b-29–34, GCST90001391–GCST90002121, GCST90274758–GCST90274848, and GCST004420–GCST004460; cis-eQTL data from the eQTLGen consortium (https://www.eqtlgen.org/ (accessed on 1 November 2026)). The Fu-iCCA cohort proteogenomic data were originally published by Dong et al. Supplementary Figures S1–S7 and Supplementary Tables S1–S22 are provided in the revision package. The MR instrumental-variable table, sensitivity analyses and mediation estimates are provided in Supplementary Tables S1–S22. The MR and single-cell analysis code is provided as Supplementary Code S1; a permanent archive will be deposited in Zenodo upon acceptance. A computational proof-of-concept is provided in Supplementary Computational Note S1 and Supplementary Figure S7; it does not constitute experimental validation. Original, uncropped Western blot images (including all blots shown in Figure 1G, Figure 2A, Figure 3I,M and Figure 4F,G,I–L) are provided as Original gel files. All other raw data are available from the corresponding author upon reasonable request, subject to institutional policies.

## Supplementary Materials

The following supporting information can be downloaded at: https://www.mdpi.com/article/10.3390/cells15212039/s1, Supplementary Figure S1: Mendelian randomization sensitivity analyses (A, forest plot; B, MHC-region analysis; C, mediation estimates; D, leave-one-out analysis); Supplementary Figure S2: secondary cis-eQTL Mendelian randomization of the nine candidate genes; Supplementary Figure S3: Kaplan–Meier survival analysis of the nine candidate genes; Supplementary Figure S4: pan-cancer AP1S3 expression; Supplementary Figure S5: AP1S3 overexpression validation; Supplementary Figure S6: corrected HER2 EIPDLL motif and SASA; Supplementary Figure S7: computational proof-of-concept. Supplementary Table S1: MR instrument variables; Supplementary Table S2: MR sensitivity analyses; Supplementary Table S3: mediation estimates; Supplementary Table S4: sample overlap; Supplementary Table S5: confounder definitions; Supplementary Table S6: STROBE-MR checklist; Supplementary Table S7: AP1S3 cis-eQTL MR; Supplementary Table S8: TCGA-CHOL Cox analysis; Supplementary Table S9: AP1S1/AP1S2/AP1S3 isoform comparison; Supplementary Table S10: multiplicity inventory; Supplementary Table S11: selection criteria; Supplementary Table S12: cytokine dose justification; Supplementary Table S13: AP-1/mutant controls; Supplementary Table S14: cell annotation; Supplementary Table S15: xenograft design; Supplementary Table S16: computational reproducibility; Supplementary Table S17: statistical reporting; Supplementary Table S18: clinical cohort characteristics; Supplementary Table S19: cell-line characteristics; Supplementary Table S20: cell-line panel; Supplementary Table S21: ADMET numeric values; Supplementary Table S22: primers and antibodies. Supplementary Code S1: MR and single-cell analysis scripts. Supplementary Computational Note S1: computational proof-of-concept.

## Abbreviations

The following abbreviations are used in this manuscript:

ADMET	Absorption, Distribution, Metabolism, Excretion, Toxicity
AP1S3	Adaptor Related Protein Complex 1 Subunit Sigma 3
BCA	Bicinchoninic Acid Assay
CCA	Cholangiocarcinoma
CHX	Cycloheximide
CI	Confidence Interval
Co-IP	Co-immunoprecipitation
CQ	Chloroquine
DAPI	4′,6-Diamidino-2-Phenylindole
DEG	Differentially Expressed Gene
DMEM	Dulbecco’s Modified Eagle Medium
DTT	Dithiothreitol
DSP	Dithiobis (succinimidyl propionate)
ECD	Extracellular Domain
EdU	5-Ethynyl-2′-deoxyuridine
ECL	Enhanced Chemiluminescence
EGFR	Epidermal Growth Factor Receptor
FBS	Fetal Bovine Serum
GEO	Gene Expression Omnibus
GSEA	Gene Set Enrichment Analysis
GWAS	Genome-Wide Association Study
HER2/ERBB2	Human Epidermal Growth Factor Receptor 2
HRP	Horseradish Peroxidase
ICD	Intracellular Domain
IDR	Intrinsically Disordered Region
IF	Immunofluorescence
IL-17A	Interleukin-17A
IVW	Inverse Variance Weighted
LLM	Large Language Model
logD	Distribution Coefficient
logP	Partition Coefficient
logS	Aqueous Solubility
MD	Molecular Dynamics
MesNa	Sodium 2-Mercaptoethanesulfonate
MR	Mendelian Randomization
MW	Molecular Weight
NF-κB	Nuclear Factor Kappa-B
nHA	Number of Hydrogen Bond Acceptors
nHD	Number of Hydrogen Bond Donors
nHet	Number of Heteroatoms
nRing	Number of Rings
nRot	Number of Rotatable Bonds
oeAP1S3	Overexpression Plasmid of AP1S3
OR	Odds Ratio
PCA	Principal Component Analysis
PPI	Protein–Protein Interaction
PVDF	Polyvinylidene Fluoride
RELA	RELA Proto-Oncogene, NF-κB Subunit (p65)
Rg	Radius of Gyration
RMSD	Root Mean Square Deviation
RMSF	Root Mean Square Fluctuation
RPMI-1640	Roswell Park Memorial Institute 1640 Medium
RT-qPCR	Quantitative Real-Time Polymerase Chain Reaction
SASA	Solvent-Accessible Surface Area
scRNA-seq	Single-Cell RNA Sequencing
SDS-PAGE	Sodium Dodecyl Sulfate-Polyacrylamide Gel Electrophoresis
sh-AP1S3	Short Hairpin RNA Targeting AP1S3
TCGA	The Cancer Genome Atlas
TM	Transmembrane Domain
TNF-α	Tumor Necrosis Factor-α
TPSA	Topological Polar Surface Area
t-SNE	t-Distributed Stochastic Neighbor Embedding
UMAP	Uniform Manifold Approximation and Projection
OS	Overall Survival
DFS	Disease-Free Survival
MSigDB	Molecular Signatures Database
