# Supplementary material for: AP1S3 Promotes Proliferation and Invasion of Cholangiocarcinoma Cells and Modulates HER2 Trafficking
Source: Cells. 2026 Nov 9;15(21):2039. doi: 10.3390/cells15212039 (PMC13761627; doi:10.3390/cells15212039)
Supplement: Supplementary file 1 [file cells-15-02039-s001.zip › cells-4651671-supplementary.pdf]

Supplementary Materials

AP1S3 Promotes Proliferation and Invasion of Cholangiocarcinoma Cells and Modulates HER2 Trafficking

Contents: Supplementary Figures S1–S7; Supplementary Tables S1–S22; Supplementary Code S1; Supplementary Computational Note S1.

All captions and file names are in English. Tables are also provided as CSV files with matching names.

List of Supplementary Items

Item	Caption
Supplementary Figure S1	Mendelian randomization sensitivity analyses. (A) Forest plot of the strict inverse-variance-weighted (IVW) estimate (8 IVs; MHC excluded). (B) MHC-region sensitivity analysis. (C) Exploratory two-step MR mediation estimates. (D) Leave-one-out sensitivity analysis for the strict IVW estimate.
Supplementary Figure S2	Secondary cis-eQTL Mendelian randomization of the nine candidate genes. Forest plots show the IVW estimates for the nine genes prioritized from the 553-gene screen. These analyses were exploratory and nominal P values were used only for prioritization.
Supplementary Figure S3	Kaplan–Meier survival analysis of the nine candidate genes in the Fu-iCCA cohort. Curves compare high versus low expression for each gene. These analyses are exploratory and not adjusted for multiple testing.
Supplementary Figure S4	Pan-cancer AP1S3 expression across TCGA tumour and normal tissues. Box plots show AP1S3 expression (log2 TPM) in tumour and adjacent normal samples. This comparison is descriptive.
Supplementary Figure S5	AP1S3 overexpression validation in HuCCT1 and QBC939 cells. (A) Western blot and qPCR validation of AP1S3 overexpression. (B) Colony formation. (C) EdU incorporation. (D) CCK-8 proliferation. (E,F) Migration and invasion assays. Data are from three biological replicates.
Supplementary Figure S6	Corrected HER2 EIPDLL motif and per-residue solvent-accessible surface area (SASA). The EIPDLL motif is at residues 930–935 within the HER2 kinase domain, not at 1001–1015 and not in the C-terminal intrinsically disordered region. SASA was calculated on HER2 kinase structure 3PP0; I931 and L935 are largely buried. Accessibility was not tested experimentally.
Supplementary Figure S7	Computational proof-of-concept for targeting the candidate AP1S3–HER2 interface. (A) Virtual screening and in silico optimization workflow. (B) Docking poses and scores. (C–G) Single 100-ns MD simulations (RMSD, SASA, Rg, hydrogen bonds, RMSF). These data are computational only and are not experimental validation; the docking-score difference is within Vina error, and no replicate MD trajectories or free-energy calculations were performed.
Supplementary Table S1	Mendelian randomization instrument variables.
Supplementary Table S2	Mendelian randomization sensitivity analyses.
Supplementary Table S3	Exploratory two-step MR mediation estimates.
Supplementary Table S4	Sample overlap between exposure and outcome GWAS.
Supplementary Table S5	Pre-specified confounder definitions.
Supplementary Table S6	STROBE-MR checklist.
Supplementary Table S7	AP1S3 cis-eQTL Mendelian randomization.
Supplementary Table S8	TCGA-CHOL Cox proportional-hazards analysis.
Supplementary Table S9	AP1S1, AP1S2 and AP1S3 isoform comparison.
Supplementary Table S10	Multiplicity inventory for exploratory analyses.
Supplementary Table S11	Selection criteria for mediators and AP1S3 prioritization.
Supplementary Table S12	Cytokine dose justification.
Supplementary Table S13	AP-1 complex and mutant controls.
Supplementary Table S14	Single-cell annotation criteria.
Supplementary Table S15	Xenograft design and reporting.

Supplementary Table S16	Computational reproducibility checklist.
Supplementary Table S17	Statistical reporting summary.
Supplementary Table S18	Clinical cohort characteristics.
Supplementary Table S19	Cell-line characteristics.
Supplementary Table S20	Cell-line panel and missing models.
Supplementary Table S21	Numeric ADMET predictions.
Supplementary Table S22	Primers and antibodies.
Supplementary Code S1	MR and single-cell analysis scripts.
Supplementary Computational Note S1	Computational proof-of-concept; not experimental validation.

Supplementary Figure S1

Mendelian randomization sensitivity analyses.

(A) Forest plot of the strict inverse-variance-weighted (IVW) estimate (8 IVs; MHC excluded). (B) MHC-region sensitivity analysis. (C) Exploratory two-step MR mediation estimates. (D) Leave-one-out sensitivity analysis for the strict IVW estimate.

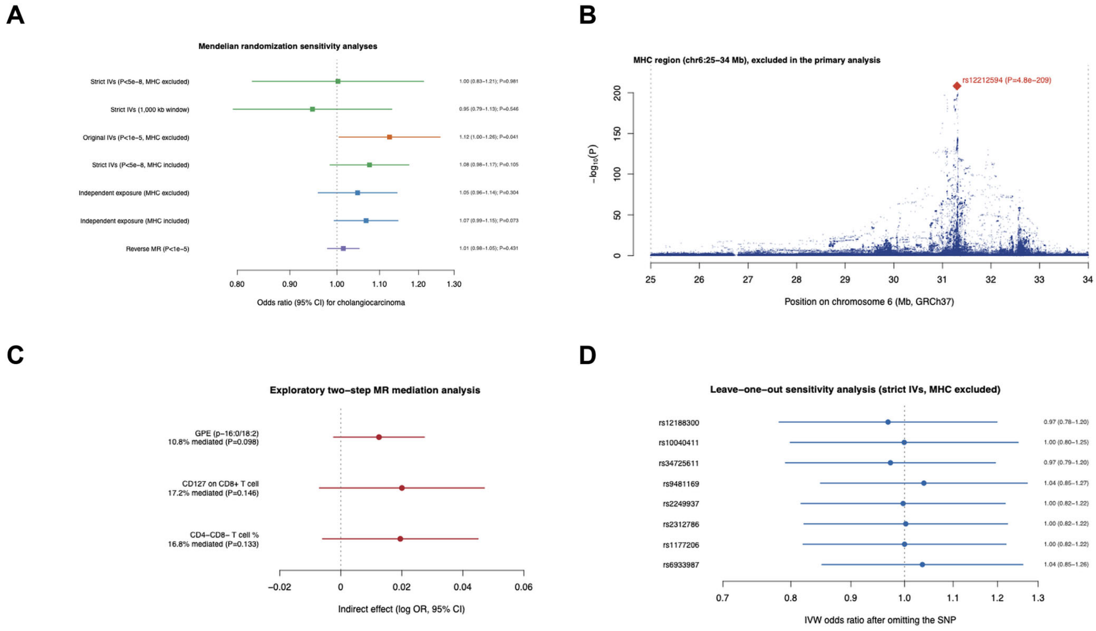

Supplementary Figure S2

Secondary cis-eQTL Mendelian randomization of the nine candidate genes.

Forest plots show the IVW estimates for the nine genes prioritized from the 553-gene screen. These analyses were exploratory and nominal P values were used only for prioritization.

A

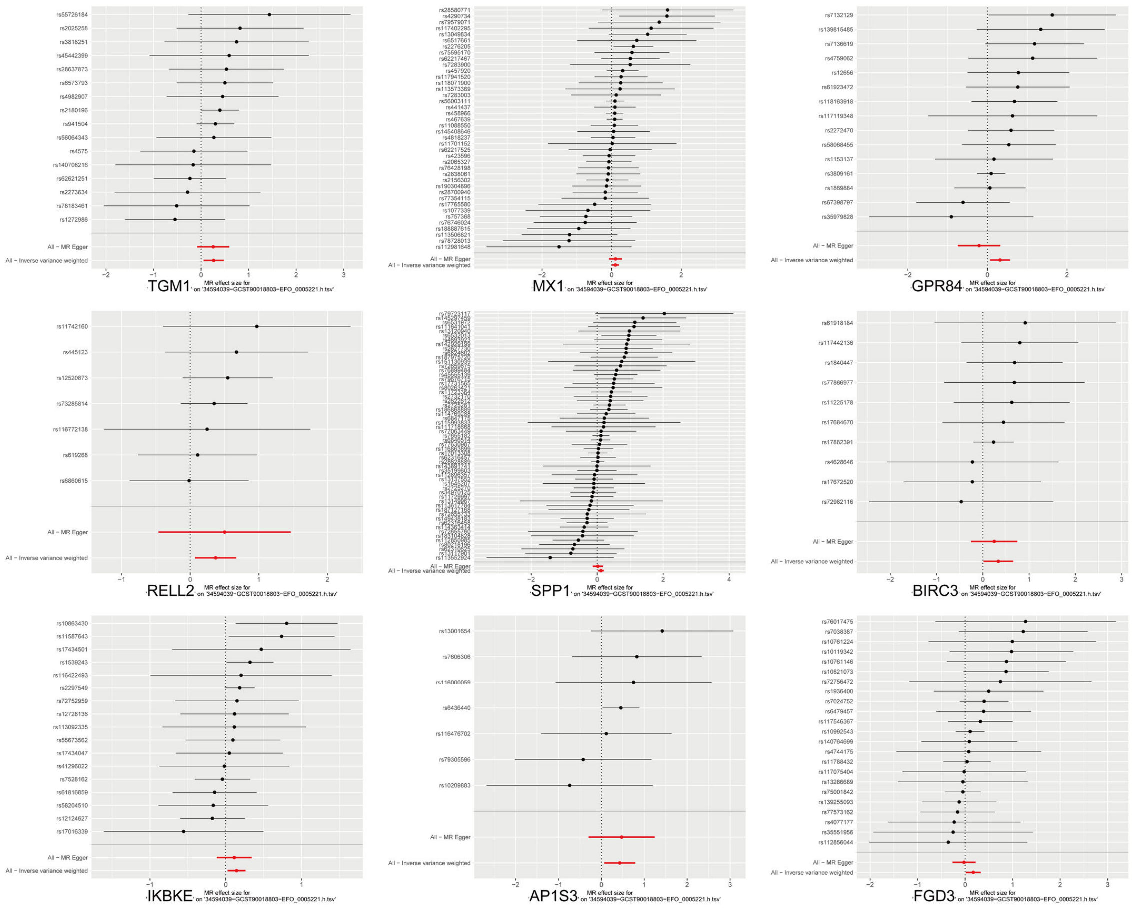

Supplementary Figure S3

Kaplan–Meier survival analysis of the nine candidate genes in the Fu-iCCA cohort.

Curves compare high versus low expression for each gene. These analyses are exploratory and not adjusted for multiple testing.

A

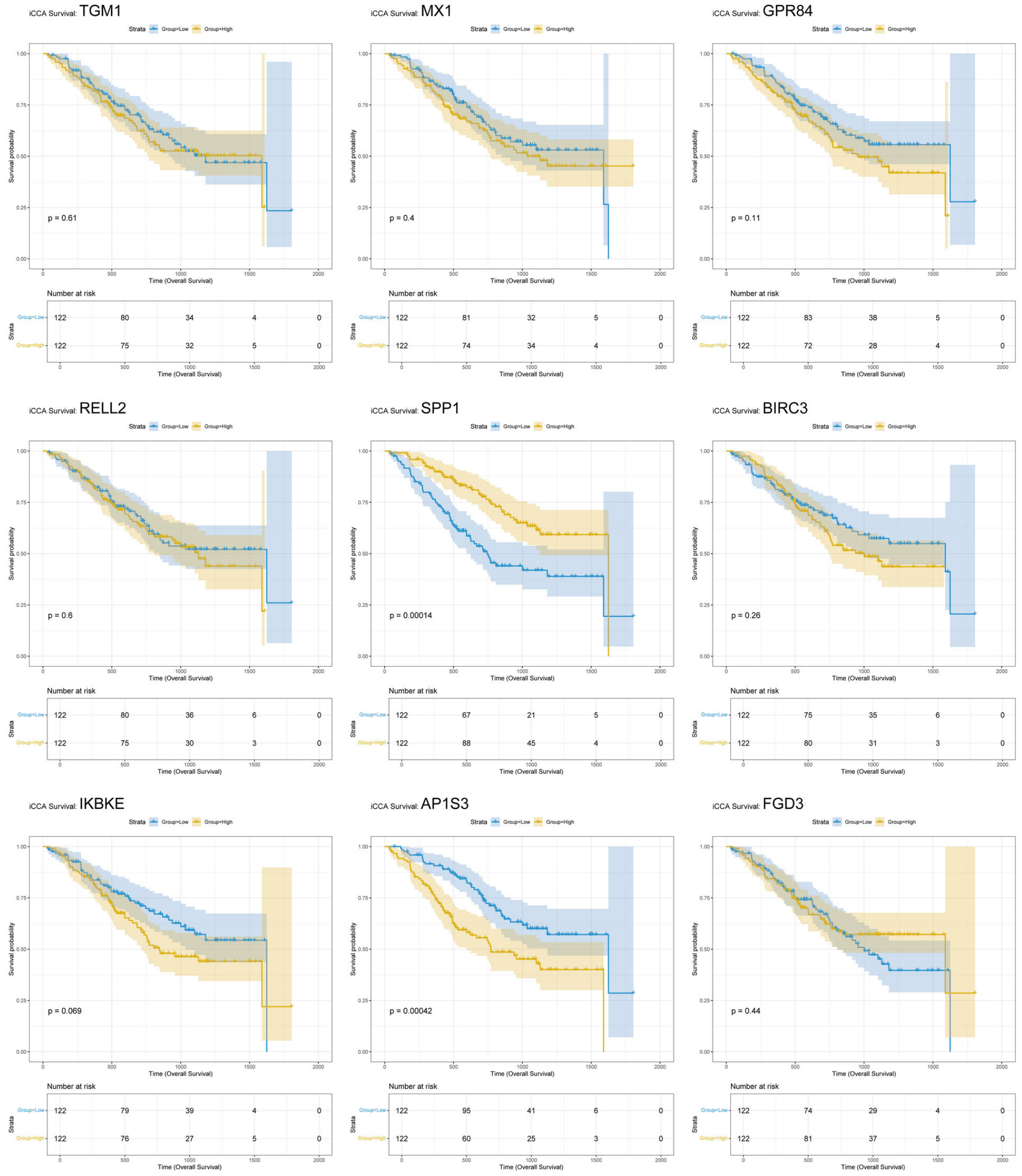

Supplementary Figure S4

Pan-cancer AP1S3 expression across TCGA tumour and normal tissues.

Box plots show AP1S3 expression (log2 TPM) in tumour and adjacent normal samples. This comparison is descriptive.

A

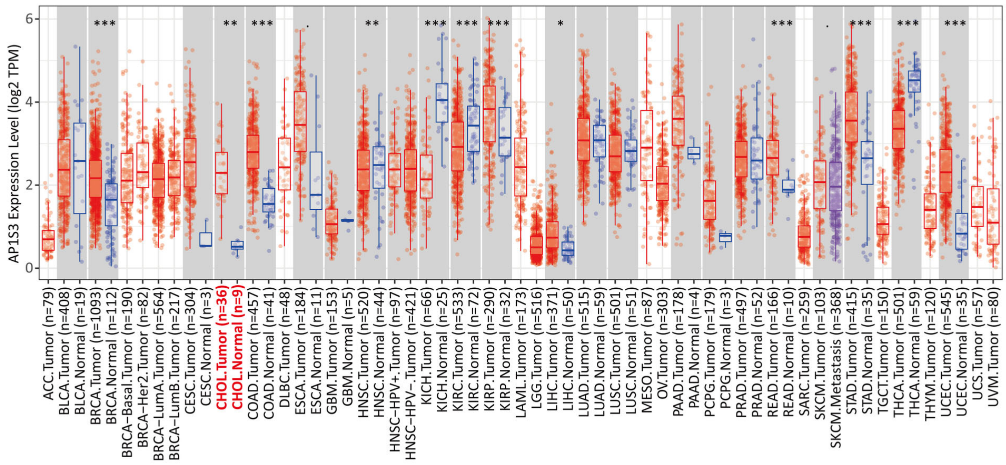

Supplementary Figure S5

AP1S3 overexpression validation in HuCCT1 and QBC939 cells.

(A) Western blot and qPCR validation of AP1S3 overexpression. (B) Colony formation. (C) EdU incorporation. (D) CCK-8 proliferation. (E,F) Migration and invasion assays. Data are from three biological replicates.

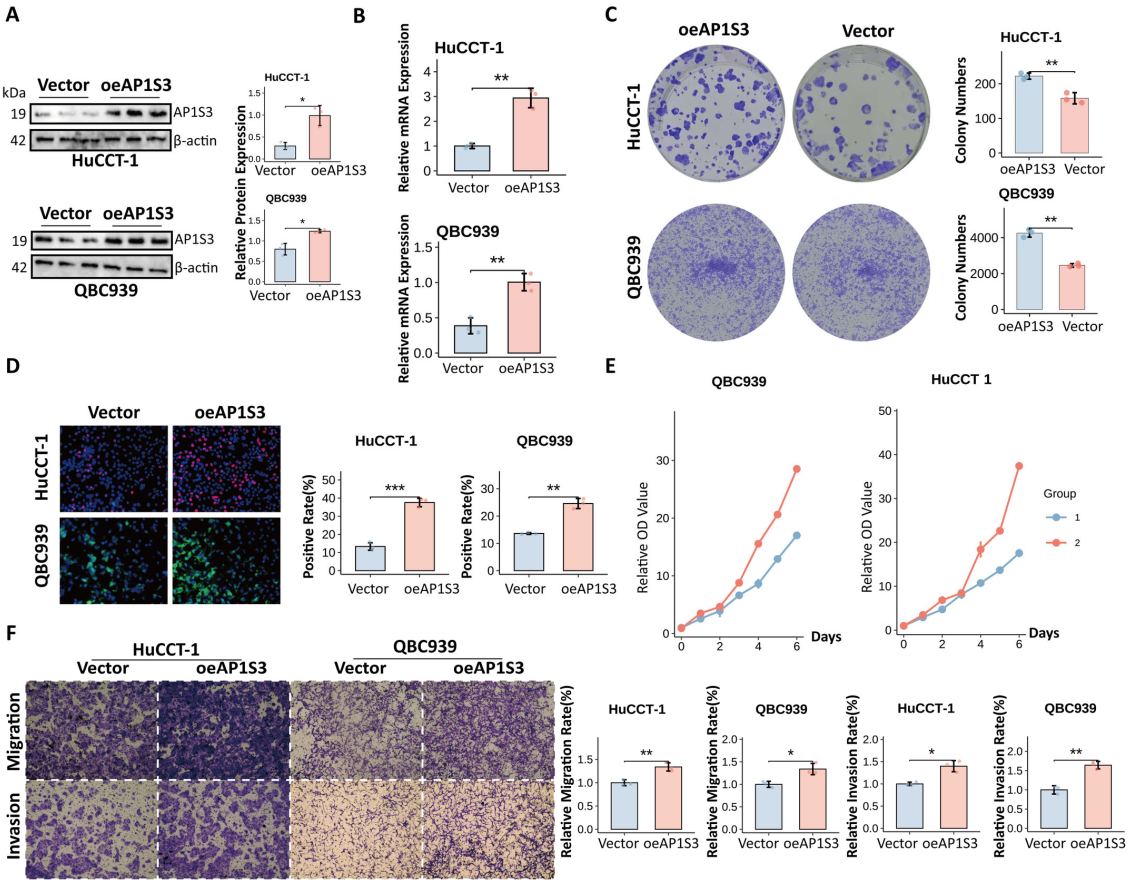

Supplementary Figure S6

Corrected HER2 EIPDLL motif and per-residue solvent-accessible surface area (SASA).

The EIPDLL motif is at residues 930–935 within the HER2 kinase domain, not at 1001–1015 and not in the C-terminal intrinsically disordered region. SASA was calculated on HER2 kinase structure 3PP0; I931 and L935 are largely buried. Accessibility was not tested experimentally.

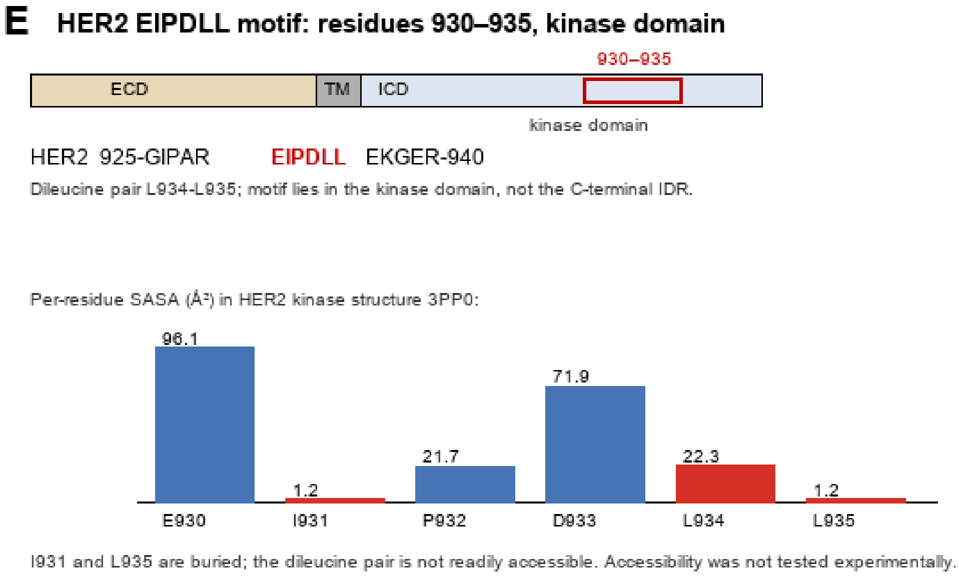

Supplementary Figure S7

Computational proof-of-concept for targeting the candidate AP1S3–HER2 interface.

(A) Virtual screening and in silico optimization workflow. (B) Docking poses and scores. (C–G) Single 100-ns MD simulations (RMSD, SASA, Rg, hydrogen bonds, RMSF). These data are computational only and are not experimental validation; the docking-score difference is within Vina error, and no replicate MD trajectories or free-energy calculations were performed.

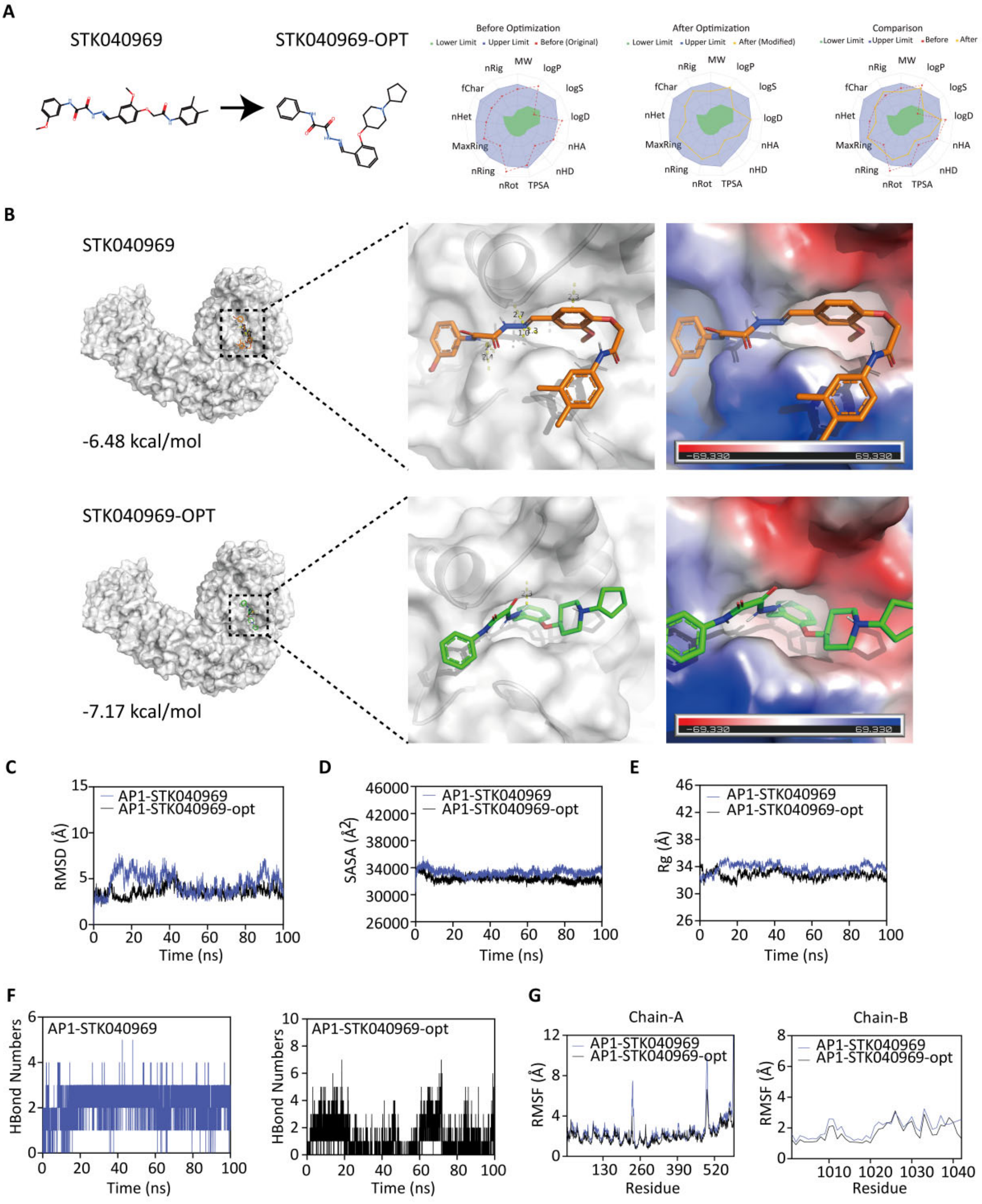

Supplementary Table S1

Mendelian randomization instrument variables.

SNP	CHR	POS	EA	OA	EAF	beta_exp	SE_exp	P_exp	F	beta_out	SE_out	P_out	Wald_OR	Wald_P	MHC	Used	Reason
rs1177206	2	61075189	T	C	0.3616	-0.1212	0.0206	3.65e-09	34.62	-0.0038	0.0436	0.9297	1.032	0.9305	False	Yes	
rs2312786	3	101633360	A	G	0.2314	0.1403	0.0229	9.61e-10	37.54	2.00e-04	0.0487	0.9965	1.001	0.9967	False	Yes	
rs10040411	5	158812417	T	C	0.3683	-0.2372	0.0297	1.45e-15	63.78	-0.0025	0.045	0.9554	1.011	0.9557	False	Yes	
rs12188300	5	158829527	T	A	0.0849	0.3952	0.0397	2.57e-23	99.1	0.057	0.0872	0.5128	1.155	0.5133	False	Yes	
rs9481169	6	111929862	T	G	0.07761	0.256	0.0373	6.58e-12	47.1	-0.0748	0.0762	0.3264	0.7466	0.3263	False	Yes	
rs6933987	6	138239434	T	C	0.2255	0.1258	0.0223	1.78e-08	31.82	-0.0697	0.0522	0.1813	0.5746	0.1818	False	Yes	
rs2249937	6	159515309	G	T	0.7685	-0.158	0.0233	1.18e-11	45.98	-0.009	0.0529	0.8643	1.059	0.8649	False	Yes	
rs34725611	19	10477067	G	A	0.3051	-0.1636	0.0225	3.80e-13	52.87	-0.0317	0.0435	0.466	1.214	0.4662	False	Yes	
rs6672420	1	25291010	T	A	0.5202	0.1237	0.0201	7.01e-10	37.87	0.0135	0.0406	0.7402	1.115	0.7395	False	No	Palindromic A/T SNP with MAF >0.42 (ambiguous)
rs3116852	6	29135647	G	A	0.5307	0.1199	0.021	1.19e-08	32.6	0.0446	0.041	0.2768	1.451	0.2767	True	No	MHC region chr6:25-34 Mb (GRCh37)
rs2734967	6	29835518	A	T	0.3074	0.1451	0.0229	2.52e-10	40.15	0.0571	0.0446	0.2006	1.482	0.2005	True	No	MHC region chr6:25-34 Mb (GRCh37)
rs12212594	6	31300819	C	T	0.05513	1.445	0.0468	4.82e-209	953.9	0.0694	0.0941	0.461	1.049	0.4608	True	No	MHC region chr6:25-34 Mb (GRCh37)
rs68094537	6	31397828	C	T	0.2838	0.3628	0.024	1.80e-51	228.5	0.026	0.0469	0.5797	1.074	0.5793	True	No	MHC region chr6:25-34 Mb (GRCh37)
rs61268343	6	32615050	C	T	0.07113	0.7148	0.0804	6.07e-19	79.04	0.1281	0.0888	0.149	1.196	0.1491	True	No	MHC region chr6:25-34 Mb (GRCh37)
rs9277195	6	33023926	T	C	0.515	-0.1215	0.0207	4.23e-09	34.45	-0.0155	0.0408	0.7044	1.136	0.704	True	No	MHC region chr6:25-34 Mb (GRCh37)

Supplementary Table S2

Mendelian randomization sensitivity analyses.

Role	Analysis	Exposure	Outcome	Threshold	Clumping_window	MHC	N_IV	OR	CI_low	CI_high	P	Q_P	Egger_intercept_P	MR_PRESSO_P	PRESSO_outliers	Weighted_median_P	F_median	F_min	Detectable_OR_80pct	Power_at_OR_1.12	Egger_OR	WM_OR	Egger_slope_P
Main	Primary: P<5e-8, MHC excluded	Sakaue 2021 (GCST90018907)	Sakaue 2021 CCA (GCST90018803)	P<5e-8	10,000 kb	Excluded	8	1.002	0.827	1.214	0.9811	0.8088	0.5399	0.7967	0	0.8814	46.5	31.8	1.315	0.211	1.135	1.026	0.558
Sensitivity	Sensitivity: 1,000 kb clumping window	Sakaue 2021 (GCST90018907)	Sakaue 2021 CCA (GCST90018803)	P<5e-8	1,000 kb	Excluded	9	0.947	0.793	1.131	0.5461	0.6338	0.8829	0.6333	0	0.9272	47.1	31.8	1.289	0.239	0.917	1.016	0.71
Sensitivity	Sensitivity: 250 kb clumping window	Sakaue 2021 (GCST90018907)	Sakaue 2021 CCA (GCST90018803)	P<5e-8	250 kb	Excluded	9	0.947	0.793	1.131	0.5461	0.6338	0.8829	0.6333	0	0.9272	47.1	31.8	1.289	0.239	0.917	1.016	0.71
Reconstructed original	Primary re-analysis: P<1e-5, MHC excluded	Sakaue 2021 (GCST90018907)	Sakaue 2021 CCA (GCST90018803)	P<1e-5	10,000 kb	Excluded	50	1.125	1.005	1.26	0.04113	0.3155	0.5007	0.31	1	0.925	24.5	19.6	1.175	0.502	1.233	1.035	0.157
Sensitivity	Sensitivity: MHC included	Sakaue 2021 (GCST90018907)	Sakaue 2021 CCA (GCST90018803)	P<5e-8	10,000 kb	Included	14	1.076	0.985	1.175	0.1055	0.901	0.8368	0.9233	0	0.7318	46.5	31.8	1.134	0.712	1.068	1.054	0.18
Replication	Independent exposure: strict, MHC excluded	Stuart 2021 (GCST90019016)	Sakaue 2021 CCA (GCST90018803)	P<5e-8	10,000 kb	Excluded	49	1.047	0.959	1.144	0.3044	0.4743	0.7264	0.4667	1	0.871	47.7	30.6	1.135	0.712	1.082	1.047	0.447
Replication	Independent exposure: 1,000 kb	Stuart 2021 (GCST90019016)	Sakaue 2021 CCA (GCST90018803)	P<5e-8	1,000 kb	Excluded	50	1.025	0.939	1.119	0.583	0.3682	0.9935	0.3967	2	0.8808	46	30.6	1.134	0.717	1.026	1.042	0.801
Replication	Independent exposure: 250 kb	Stuart 2021 (GCST90019016)	Sakaue 2021 CCA (GCST90018803)	P<5e-8	250 kb	Excluded	58	0.998	0.918	1.084	0.9553	0.3666	0.9004	0.39	2	0.9646	45.8	30.6	1.125	0.762	1.008	1.012	0.932
Replication	Independent exposure: P<1e-5	Stuart 2021 (GCST90019016)	Sakaue 2021 CCA (GCST90018803)	P<1e-5	10,000 kb	Excluded	96	1.009	0.935	1.089	0.8141	0.4999	0.7419	0.52	5	0.8953	30.6	19.7	1.115	0.83	1.036	1.045	0.69
Replication	Independent exposure: MHC included	Stuart 2021 (GCST90019016)	Sakaue 2021 CCA (GCST90018803)	P<5e-8	10,000 kb	Included	55	1.067	0.994	1.146	0.07309	0.6172	0.4684	0.65	1	0.7436	50.3	30.6	1.107	0.877	1.109	1.084	0.103
Reverse	Reverse MR: CCA to psoriasis (Sakaue outcome)	Sakaue 2021 CCA (GCST90018803)	Sakaue 2021 psoriasis	P<1e-5	10,000 kb	Excluded	19	1.014	0.979	1.051	0.4306	0.6859	0.5249	0.7067	3	0.7386	21.2	19.6	1.052	1	1.027	1.02	0.3
Reverse	Reverse MR: CCA to psoriasis (Stuart outcome)	Stuart 2021 (GCST90019016)	Stuart 2021 psoriasis	P<1e-5	10,000 kb	Excluded	15	0.996	0.967	1.026	0.7969	0.5663	0.6727	0.5867	0	0.9141	20.7	19.7	1.044	1	0.99	0.994	0.63

Role	Analysis	Exposure	Outcome	Threshold	Clumping_window	MHC	N_IV	OR	CI_low	CI_high	P	Q_P	Egger_intercept_P	MR_PRESSO_P	PRESSO_outliers	Weighted_median_P	F_median	F_min	Detectable_OR_80pct	Power_at_OR_1.12	Egger_OR	WM_OR	Egger_slope_P
Sensitivity	Sensitivity: IL12B/TYK2 loci removed	Sakaue 2021 (GCST90018907)	Sakaue 2021 CCA (GCST90018803)	P<5e-8	10,000 kb	Excluded	5	0.872	0.645	1.18	0.375									0.11			

Supplementary Table S3

Exploratory two-step MR mediation estimates.

Mediator	Mediator_GWAS	Path_a_beta	Path_a_SE	Path_b_beta	Path_b_SE	Total_c_beta	Total_c_SE	Indirect_a_x_b	Indirect_SE	Indirect_95CI	Sobel_P	Proportion_mediated_pct	Proportion_95CI_conditional_pct	Direction	Interpretation
GPE (p-16:0/18:2)	GCST90300318	-0.02802	0.01362	-0.4475	0.161	0.1165	0.05583	0.01254	0.007582	-0.002 to 0.027	0.09822	10.76	-2.4 to 66.6	a<0, b<0; lower mediator -> higher CCA risk	95% CI includes zero; exploratory only
CD127 on CD8+ T cell	GCST90001929	-0.05871	0.02914	-0.3415	0.1626	0.1165	0.05583	0.02005	0.01379	-0.007 to 0.047	0.146	17.21	-6.3 to 108.6	a<0, b<0; lower mediator -> higher CCA risk	95% CI includes zero; exploratory only
CD4-CD8- T cell %	GCST90001613	-0.05411	0.02629	-0.3608	0.1643	0.1165	0.05583	0.01952	0.013	-0.006 to 0.045	0.1331	16.76	-5.0 to 107.3	a<0, b<0; lower mediator -> higher CCA risk	95% CI includes zero; exploratory only

Supplementary Table S4

Sample overlap between exposure and outcome GWAS.

Item	Value
Psoriasis GWAS	Sakaue et al. 2021, GCST90018907; 5,072 European cases/478,102 controls; 206 East Asian cases/172,289 controls; cohorts BBJ, UKB, FinnGen
CCA GWAS	Sakaue et al. 2021, GCST90018803; 832 European cases/475,259 controls; 418 East Asian cases/159,201 controls; cohorts BBJ, UKB, FinnGen
Shared cohorts	Both traits were meta-analysed from the same three cohorts (BBJ, UKB, FinnGen).
Maximum shared controls, European	475,259 (100% of CCA controls; 99.4% of psoriasis controls)
Maximum shared controls, East Asian	159,201 (100% of CCA controls; 92.4% of psoriasis controls)
Case overlap	Different diseases; overlap expected to be small but not zero (individuals with both conditions).

Item	Value
Empirical check	Cross-trait LDSC intercept = 0.003 (SE 0.0043; P=0.485); genetic correlation = 0.284 (SE 0.248; P=0.252).
Independent replication	Stuart et al. 2021 (GCST90019016; 15,967 European cases/28,194 controls; CASP, Kiel, Genizon, PsA, WTCCC2, Exomechip, PAGE, GAPC) has no cohort overlap with UKB, FinnGen or BBJ.
Replication result	IVW OR 1.047 (95% CI 0.959-1.144), P=0.304; MHC-included OR 1.067 (0.994-1.146), P=0.073.

Supplementary Table S5

Pre-specified confounder definitions.

Trait_domain	Rationale	Action_in_revised_analysis
Smoking initiation / current smoking	Established CCA and psoriasis risk factor; potential confounder	Listed a priori; not used for unstructured filtering
Alcohol intake / alcohol use disorder	Established CCA risk factor	Listed a priori; not used for unstructured filtering
Body mass index / obesity	Established CCA risk factor; shared with psoriasis	Listed a priori; not used for unstructured filtering
Type 2 diabetes / fasting glucose	Established CCA risk factor	Listed a priori; not used for unstructured filtering
Inflammatory bowel disease (Crohn disease, ulcerative colitis)	Established PSC/CCA pathway; shared autoimmune architecture	Listed a priori; not used for unstructured filtering
Primary sclerosing cholangitis	Strongest CCA risk factor	Listed a priori; not used for unstructured filtering
Hepatitis B / hepatitis C	Established CCA risk factors	Listed a priori; not used for unstructured filtering
Cirrhosis / NAFLD / alcohol-related liver disease	Established CCA risk factors	Listed a priori; not used for unstructured filtering
Psoriasis, psoriatic arthritis, ankylosing spondylitis, rheumatoid arthritis	Autoimmune traits that index shared pleiotropy	Listed a priori; global pleiotropy tested by MR-Egger/MR-PRESSO
Age, sex, ancestry	Demographic confounders	Adjusted by study design/ancestry restriction; not SNP-level traits

Supplementary Table S6

STROBE-MR checklist.

Item	Topic	Requirement	Where_addressed
1	Title and abstract	State that MR was used and summarize the causal question	Abstract; Methods 1.1
2	Background/rationale	Explain rationale for psoriasis-CCA MR	Introduction
3	Objectives	Test whether genetic liability to psoriasis affects CCA risk	Methods 1.1
4	Study design	Two-sample MR; forward and reverse	Methods 1.1
5	Data sources	GWAS Catalog IDs, ancestry, sample sizes, overlap	Methods 1.1; Table S4
6	Instrument selection	P<5e-8; LD r2<0.001; 10,000 kb; MHC excluded; F>10	Methods 1.2; Table S1
7	Power	Report detectable OR and post-hoc power	Methods 1.6; Results 2.1

Item	Topic	Requirement	Where_addressed
8	Statistical methods	IVW, weighted median, MR-Egger, MR-PRESSO, Q, Steiger	Methods 1.3
9	Pleiotropy	MHC exclusion; Egger intercept; MR-PRESSO; IL12B/TYK2 sensitivity	Methods 1.4; Table S2
10	Reverse direction	CCA to psoriasis MR	Methods 1.5; Table S2
11	Mediation	Two-step MR; proportion and 95% CI; exploratory only	Methods 1.7; Table S3
12	Main results	Report strict and relaxed estimates; do not overstate	Results 2.1; Figure S1
13	Sensitivity	Clumping window, MHC, independent exposure	Results 2.1; Table S2
14	Limitations	Low power; overlapping controls; weak mediator instruments	Discussion
15	Data/code	Public GWAS; analysis code available	Data Availability

Supplementary Table S7

AP1S3 cis-eQTL Mendelian randomization.

Analysis	SNP	EA	OA	EAF	beta_exposure	SE_exposure	P_exposure	F	beta_outcome	SE_outcome	P_outcome	OR	CI_low	CI_high	P
Single SNP	rs10209883	A	G	0.75	0.0566	0.0092	8.87e-10	37.56	-0.0418	0.056	0.4555	0.478	0.0688	3.321	0.4554
Single SNP	rs116000059	A	C	0.0248	0.1508	0.0267	1.59e-08	31.94	0.1131	0.1399	0.4189	2.117	0.3436	13.04	0.4188
Single SNP	rs116476702	T	C	0.0239	0.2371	0.0422	1.93e-08	31.57	0.0272	0.1841	0.8825	1.122	0.2448	5.138	0.8825
Single SNP	rs13001654	T	C	0.1171	-0.0845	0.0124	1.10e-11	46.14	-0.1199	0.0714	0.093	4.131	0.7889	21.63	0.0931
Single SNP	rs6436440	A	G	0.5225	0.1847	0.008	2.85e-119	539.2	0.084	0.0403	0.0372	1.576	1.028	2.417	0.0371
Single SNP	rs7606306	T	C	0.3626	-0.0574	0.0084	6.60e-12	47.14	-0.0475	0.0443	0.2838	2.286	0.5043	10.36	0.2836
Single SNP	rs79305596	T	C	0.0876	-0.0989	0.0142	2.75e-12	48.85	0.0419	0.0804	0.6021	0.6547	0.1331	3.22	0.6023
IVW (7 SNPs)												1.534	1.069	2.201	0.0203
Weighted median												1.574	1.06	2.337	0.0244
MR-Egger												1.604	0.7419	3.469	0.2834
MR-PRESSO global test															0.628

Supplementary Table S8

TCGA-CHOL Cox proportional-hazards analysis.

Model	N	Events	Term	HR	CI_low	CI_high	P	LR_interaction_P
Unadjusted	33	18	AP1S3_z	0.8896	0.5487	1.442	0.6352	
Unadjusted	33	18	ERBB2_z	1.636	0.6361	4.21	0.307	

Model	N	Events	Term	HR	CI_low	CI_high	P	LR_interaction_P
Unadjusted	33	18	AP1S3_z:ERBB2_z	2.108	0.952	4.668	0.06595	0.0285
Age + sex	33	18	AP1S3_z	0.7749	0.4444	1.351	0.3685	
Age + sex	33	18	ERBB2_z	1.731	0.6449	4.648	0.276	
Age + sex	33	18	AP1S3_z:ERBB2_z	2.198	0.927	5.212	0.07379	0.03089
Age + sex + stage	23	8	AP1S3_z	0.8313	0.2913	2.372	0.7298	
Age + sex + stage	23	8	ERBB2_z	1.473	0.5441	3.985	0.4461	
Age + sex + stage	23	8	AP1S3_z:ERBB2_z	1.623	0.6326	4.163	0.3138	0.2304
Subgroup: ERBB2-low (continuous)	17	10	AP1S3_z	0.5768	0.2398	1.388	0.2192	
Subgroup: ERBB2-high (continuous)	16	8	AP1S3_z	1.563	0.6268	3.899	0.338	
Log-rank: ERBB2-low	17	10	AP1S3 high vs low				0.3415	
Log-rank: ERBB2-high	16	8	AP1S3 high vs low				0.122	

Supplementary Table S9

AP1S1, AP1S2 and AP1S3 isoform comparison.

Isoform	UniProt	Length	Identity_to_AP1S3_pct	TCGA_CHOL_median_log2	Fu_iCCA_median_log2
AP1S1 (σ1A)	P61966	158	69.5	11.35	4.95
AP1S2 (σ1B)	P56377	157	73.2	9.28	5.3
AP1S3 (σ1C)	Q96PC3	154	100	8.58	4.37

Supplementary Table S10

Multiplicity inventory for exploratory analyses.

Analysis	Number_of_tests	Correction	Threshold_or_q	Result	Interpretation
Primary psoriasis→CCA (strict)	1	None (single pre-specified hypothesis)	0.05	IVW OR 1.002 (0.827–1.214); P=0.981	Inconclusive; no causal claim
Original relaxed P<1e-5	1	Bonferroni across 659 exploratory tests	7.6×10^{-5}	IVW OR 1.125 (1.005–1.260); P=0.041	Would not survive correction
Secondary cis-eQTL MR (553 genes)	553	Bonferroni / nominal prioritization	9.0×10^{-5}	AP1S3 P=0.020; rs6436440-driven	Not significant after correction; candidate only
Mediation (3 mediators)	3	Benjamini–Hochberg FDR	q=0.146	All Sobel P>0.09; all CIs include zero	Not significant

Analysis	Number_of_tests	Correction	Threshold_or_q	Result	Interpretation
Sensitivity analyses (MHC, window, independent exposure, reverse)	—	Not independent primary tests	—	Strict OR 1.002; independent OR 1.047; reverse OR 1.014	Support inconclusive interpretation

Supplementary Table S11

Selection criteria for mediators and AP1S3 prioritization.

Step	Criterion	Number_retained	Threshold	Interpretation
Step 1: DEG intersection	GSE54456 psoriasis lesion vs normal; TCGA-CHOL tumour vs normal; log2FC>1 and FDR<0.05 in both	553	FDR<0.05	Discovery set; not a mediation test
Step 2: secondary cis-eQTL MR	eQTLGen cis-eQTL instruments; nominal P<0.05 for prioritization	9	P<0.05 (nominal)	Exploratory; no FDR control
Step 3: survival filter	Fu-iCCA cohort; P<0.05 and HR>1	1 (AP1S3)	P<0.05	Data-driven sequential filter; independent cohort but selection bias possible
Step 4: AP1S3 cis-eQTL MR	7 SNPs; IVW OR 1.53 (1.07–2.20), P=0.020	1	Bonferroni 9 genes: 0.0056; 553 genes: 9.0×10^{-5}	Not significant after correction; candidate only
Mediator screen	Path a and path b both nominal P<0.05; 3 mediators	3	P<0.05	BH-FDR q=0.146; all indirect CIs include zero

Supplementary Table S12

Cytokine dose justification.

Agent	Dose_used	Typical_circulating_level	Approx_fold_above_serum	Rationale	Limitation
IL-17A	100 ng/mL	Low pg/mL to tens of pg/mL in serum	~10^3–10^4-fold	Prior mechanistic studies in CCA/keratinocytes; robust NF-κB activation in 24–48 h	Supraphysiologic; no dose-response; no single-cytokine control
TNF-α	50 ng/mL	Low pg/mL to tens of pg/mL in serum	~10^2–10^3-fold	Same as above	Supraphysiologic; combination may activate multiple pathways
IL-17A + TNF-α	100 + 50 ng/mL	—	—	Synergistic NF-κB activation	Cannot attribute effect to one cytokine; no physiological validation
Lower doses / patient fluids	0.1–10 ng/mL; 5–10% serum/conditioned medium	—	—	Proposed validation	Not performed in this revision

Supplementary Table S13

AP-1 complex and mutant controls.

Control_experiment	Design	Purpose
AP1S3 vs AP1S1/AP1S2	Separate knockdown/rescue of each σ1 isoform; HER2 surface/recycling	Test isoform specificity
AP-1 complex	AP-1 γ/β knockdown; native PAGE/Co-IP for complex integrity	Test hemicomplex requirement
Mutant control	EIPDAA vs WT EIPDLL vs control mutation outside motif	Exclude general folding effect
AP1S3-null background	HER2-WT vs HER2-EIPDAA in AP1S3 knockout; surface/total/degradation/phosphorylation	Directly test motif dependence
Localization	HER2-GFP with LAMP1, EEA1, Rab4, Rab11, CD63; live imaging/quantitative colocalization	Distinguish lysosome vs recycling endosome
Ubiquitination	HER2 K48/K63 ubiquitination ± AP1S3; proteasome/lysosome inhibitors	Define sorting mechanism
Surface/recycling controls	Internalization, MesNa efficiency, total surface normalization	Make recycling rate interpretable

Supplementary Table S14

Single-cell annotation criteria.

Lineage_or_subtype	Markers_or_method	Definition	Validation_status
Epithelial	EPCAM, KRT19, KRT8, KRT18	Canonical epithelial markers	Marker-based; no pathology review
Immune	PTPRC, CD3D, CD68	Pan-immune/T/NK/myeloid	Marker-based
Fibroblast	COL1A1, COL3A1, DCN	Fibroblast/CAF	Marker-based
Endothelial	PECAM1, VWF	Endothelial	Marker-based
Malignant epithelial	Epithelial markers + inferCNV	CNV inference	Computational; no FISH/pathology confirmation
SCEL+ squamous	SCEL, KRT13, KRT16, KRT6A	Squamous/keratinization program	Cluster association; not a validated subtype
Independent validation	TCGA-CHOL and Fu-iCCA bulk expression/survival	Independent cohorts for association	Bulk, not single-cell/IHC; no direct validation

Supplementary Table S15

Xenograft design and reporting.

Item	Design_or_status	Limitation
Model	HuCCT1 subcutaneous xenograft; 4-week-old female BALB/c nude mice	Immunodeficient; no systemic psoriasis inflammation
Groups	shNC; shAP1S3; shAP1S3 + Flag-HER2	3 groups
Animal numbers	n=5 per group; 15 total	No a priori power calculation
Allocation	Grouped after tumour formation; randomization method not documented	Selection bias possible

Item	Design_or_status	Limitation
Blinding	Tumour measurement and analysis not documented as blinded	Detection bias possible
HER2 reconstitution	Exogenous Flag-HER2 overexpression; not CRISPR knock-in; not titrated to endogenous level	May exceed physiological HER2; rescue = compensation
Tumour volume	Measured every 3 days; V=0.5×length×width²	Two-way ANOVA (time×group) with Bonferroni in Methods
Endpoint	Tumour weight after sacrifice; IF for HER2/p-HER2	One-way ANOVA/Tukey in Methods
Limitations	One cell line; subcutaneous; no orthotopic/immunocompetent model	Does not model psoriasis systemic inflammation

Supplementary Table S16

Computational reproducibility checklist.

Item	Available_or_status	Missing_or_limitation
Compound identity	STK040969 and STK040969-opt; SMILES printed in ADMETlab reports	SMILES not independently verified; no atom-level mapping to optimization steps
Chemical synthesis	Not performed	No purity, NMR, MS or batch record
Receptor structure	AP1S3 model used for docking; PDB/model source not recorded	Receptor preparation/protonation not documented
Docking	DrugCLIP and Vina scores; -6.48 vs -7.17 kcal/mol	Docking box, exhaustiveness, protonation and software versions not fully recorded; 0.7 kcal/mol within Vina error
MD	GROMACS 2022; one 100 ns trajectory per complex	Force field, water/ions, thermostat/barostat, seeds and replicates not recorded
Free energy	Not performed	No MM-PBSA/GBSA or absolute binding free energy
ADMET	ADMETlab 3.0 predictions	ML predictions; no experimental solubility, PPB, CYP, hERG or Caco-2
Pocket validation		No mutagenesis or EIPDLL competition
Selectivity		AP-1 is a shared adaptor; broad off-target trafficking possible
Experimental validation		No synthesis, SPR/ITC, cellular activity or PK

Supplementary Table S17

Statistical reporting summary.

Analysis	n	Effect_size (95% CI)	Exact_P	Test	Multiplicity
MR strict (psoriasis→CCA)	8 IVs	OR 1.002 (95% CI 0.827–1.214)	P=0.981	IVW; F median 46.5, min 31.8	Single pre-specified test; no correction
MR original P<1e-5	50 IVs	OR 1.125 (95% CI 1.005–1.260)	P=0.041	IVW	Would not survive 659-test Bonferroni (7.6e-5)
MR independent exposure	49 IVs	OR 1.047 (95% CI 0.959–1.144)	P=0.304	IVW	Replication; no correction
Reverse MR	19 IVs	OR 1.014 (95% CI 0.979–1.051)	P=0.431	IVW	Underpowered

Analysis	n	Effect_size (95% CI)	Exact_P	Test	Multiplicity
AP1S3 cis-eQTL MR	7 SNPs	OR 1.534 (95% CI 1.069–2.201)	P=0.020	IVW; F median 46.1, min 31.6	Fails Bonferroni 9/553 genes
Mediation GPE	—	10.8% (95% CI −2.4 to 66.6)	Sobel P=0.098	Two-step MR; a×b/c	BH q=0.146
Mediation CD127 on CD8+ T	—	17.2% (95% CI −6.3 to 108.6)	Sobel P=0.146	Two-step MR	BH q=0.146
Mediation CD4−CD8− T	—	16.8% (95% CI −5.0 to 107.3)	Sobel P=0.133	Two-step MR	BH q=0.146
TCGA-CHOL AP1S3 Cox	33 tumours; 18 deaths	HR 0.83 (95% CI 0.29–2.37)	P=0.73	Cox proportional hazards	Exploratory; no correction
Figure 5A HER2-high log-rank	33 tumours	—	P=0.12	Kaplan–Meier/log-rank	Subgroup; interaction P=0.066 unadjusted, 0.314 stage-adjusted
Figure 5A HER2-low log-rank	33 tumours	—	P=0.34	Kaplan–Meier/log-rank	Subgroup
Figure 1I vascular invasion	7 paired cases	—	P=0.052	Paired test	Descriptive; not adjusted
Figure 2 knockdown panels	3 biological replicates	Mean differences in Prism source files	Exact P in Prism source files	One-way ANOVA + Dunnett vs shared shNC	Dunnett correction for 2 shAP1S3 groups
CCK-8 (Figures 2E, 5C)	3 biological; 5 technical wells per experiment	Technical wells averaged before analysis	Exact P in Prism source files	One-way/two-way ANOVA	Technical wells not counted as n
Xenograft growth (Figure 5E)	5 mice/group; 3 groups	Growth curves in Prism source files	Exact P in Prism source files	Two-way repeated-measures ANOVA (time × group; mouse as subject)	Endpoint tumour weight secondary one-way ANOVA
Other in vitro panels	n=3 biological unless stated	Mean differences in Prism source files	Exact P in Prism source files	t-test/ANOVA	Tukey/Bonferroni; asterisks in figures

Supplementary Table S18

Clinical cohort characteristics.

Characteristic	Value	Note
Cohort size	7 patients	Paired tumour and adjacent normal tissue
Centre	Single centre (Second Xiangya Hospital)	Convenience sample; consecutive enrollment not recorded
Diagnosis	Pathologically confirmed CCA	All 7 cases
Specimen	Paired tumour/adjacent normal	Adjacent normal distance/matching not recorded
Age	Not available in de-identified dataset	Cannot assess age distribution
Sex	Not available	Cannot assess sex distribution
Tumour location	Not available	Intrahepatic vs extrahepatic not recorded
Histology/differentiation	Not available	Subtype/grade not recorded
TNM stage	Not available	Stage distribution unknown
HBV/HCV/cirrhosis	Not available	Confounding not assessable
Psoriasis history	Not available	No psoriasis subgroup
Power	No a priori calculation	Descriptive only; underpowered for subgroup analyses

Characteristic	Value	Note
Analysis	Paired descriptive comparison	No adjustment for confounders

Supplementary Table S19

Cell-line characteristics.

Cell_line	Origin	Source	Authentication	AP1S3	HER2_protein	HER2_amplification	Rationale	Limitation
HuCCT1	Human intrahepatic cholangiocarcinoma	Cell Bank of Chinese Academy of Sciences	STR-authenticated; mycoplasma-free	Detectable; knockdown validated	Detectable by Western blot (Figure 4L)	Not determined (no FISH/qPCR/CNV)	Established CCA line; lentivirally transducible; intrahepatic background	HER2 expression not quantified; amplification unknown
QBC939	Human bile duct cholangiocarcinoma	Guangzhou Jennio Biotech	STR-authenticated; mycoplasma-free	Detectable; knockdown validated	Detectable by Western blot (Figure 4L)	Not determined (no FISH/qPCR/CNV)	Second CCA line; independent genetic background	HER2 expression not quantified; amplification unknown
293T	Human embryonic kidney	Not a CCA line	STR-authenticated; mycoplasma-free	Not applicable	Not applicable	Not applicable	Lentiviral packaging and Co-IP only	Not a CCA model; not used for HER2 status

Supplementary Table S20

Cell-line panel and missing models.

Cell_model	Origin	Source_or_status	Used_in_study	HER2_status	Rationale_or_gap
HuCCT1	Intrahepatic CCA	Cell Bank of Chinese Academy of Sciences, Shanghai	Used	HER2 protein detectable; amplification not determined	Established intrahepatic model; lentivirally transducible
QBC939	Bile duct/extrahepatic CCA	Guangzhou Jennio Biotech, Guangzhou	Used	HER2 protein detectable; amplification not determined	Second CCA background; not equivalent to HuCCT1
293T	Human embryonic kidney	Guangzhou Jennio Biotech, Guangzhou	Used for packaging/Co-IP	Not applicable	Not a CCA model
H69	Non-malignant human cholangiocyte	Not used	Not used	Not determined	Would provide non-malignant control
HIBEC	Human intrahepatic biliary epithelial cells	Not used	Not used	Not determined	Would provide non-malignant control
RBE	Human intrahepatic CCA	Not used	Not used	Not determined	Additional CCA line
HuH28	Human extrahepatic CCA	Not used	Not used	Not determined	Additional CCA line
TFK-1	Human extrahepatic CCA	Not used	Not used	Not determined	Additional CCA line
Patient-derived organoids	Primary CCA	Not used	Not used	Not determined	Would improve clinical relevance

Supplementary Table S21

Numeric ADMET predictions.

Property	STK040969	STK040969_opt	Method	Caveat

Property	STK040969	STK040969_opt	Method	Caveat
Molecular weight	504.20	434.23	ADMETlab 3.0	Predicted; no experimental validation
logP	3.76	3.24	ADMETlab 3.0	Predicted
logS	-5.688	-3.972	ADMETlab 3.0	Predicted; higher logS = more soluble
TPSA (Å²)	127.35	83.03	ADMETlab 3.0	Predicted
H-bond donors	3	2	ADMETlab 3.0	Predicted
H-bond acceptors	10	7	ADMETlab 3.0	Predicted
Rotatable bonds	13	9	ADMETlab 3.0	Predicted
Plasma protein binding (%)	98.1	87.8	ADMETlab 3.0 / manuscript	Predicted
Free fraction (%)	1.9	12.2	Calculated from PPB	Predicted
Caco-2 permeability	Not reported numerically	Not reported numerically	ADMETlab 3.0	Predicted; direction only
hERG toxicity	Not reported numerically	Not reported numerically	ADMETlab 3.0	Predicted; direction only
CYP inhibition	Not reported numerically	Not reported numerically	ADMETlab 3.0	Predicted; direction only

Supplementary Table S22

Primers and antibodies.

Reagent	Vendor	Application	Caveat
AP1S3 antibody	Proteintech	WB, IF	17365-1-AP
HER2 antibody	Proteintech	WB, IF	18299-1-AP
p-HER2 antibody	Proteintech	WB, IF	82762-11-PBS
β-actin antibody	Proteintech	WB	66009-1-Ig
GAPDH antibody	Proteintech	WB	66004-1-Ig
AP1S3 primers	Sangon Biotech	RT-qPCR	AP1S3-F:TGGAAATGTCTGTGAGCTGGA;AP1S3-R:GGATGGGAGGCGTTGCTTAT
GAPDH primers	Sangon Biotech	RT-qPCR	GAPDH-F:AGAAAAACCTGCCAAATATGATGAC;GAPDH-R:TGGGTGTCGCTGTTGAAGTC

Supplementary Computational Note S1

This note describes the computational proof-of-concept for targeting the candidate AP1S3–HER2 interface. It is not experimental validation.

Virtual screening used DrugCLIP, followed by structure-guided optimization. Docking scores were −6.48 kcal/mol for STK040969 and −7.17 kcal/mol for STK040969-opt; the 0.7 kcal/mol difference is within Vina scoring error. A single 100-ns MD trajectory per complex was run in GROMACS 2022; no replicates or MM-PBSA/GBSA calculations were performed. ADMET values are ADMETlab 3.0 predictions, not experimental measurements.

The compound has not been synthesized and has not been tested by SPR, ITC, pull-down, cellular assays or pharmacokinetics. The binding pocket was not validated by mutagenesis, and competition with the HER2 EIPDLL motif was not demonstrated. AP-1 is a shared adaptor, so broad off-target trafficking effects are possible.

See Supplementary Figure S7 and Supplementary Table S21 for the computational panels and numeric ADMET values. The ADMETlab reports are provided as supplementary files.

Supplementary Code S1

The following English-named scripts are provided in Supplementary_Code_S1.zip:

- mr_lib.py
- plot_forest.R
- plot_mhc.R
- MR_analysis.R
- single_cell_analysis_wrapper.R
- single_cell_gene_plots.R

File inventory: Supplementary_Figure_S1.png–S7.png; Supplementary_Table_S1.csv–S22.csv; Supplementary_Code_S1.zip; Supplementary_Materials.docx.
