# Supplementary material for: AP1S3 Promotes Proliferation and Invasion of Cholangiocarcinoma Cells and Modulates HER2 Trafficking
Source: Cells. 2026 Nov 9;15(21):2039. doi: 10.3390/cells15212039 (PMC13761627; doi:10.3390/cells15212039)
Supplement: Supplementary file 1 [file cells-15-02039-s001.zip › cells-4651671 Supplementary_Materials_Package 2.zip!/Supplementary_Materials_Package/Supplementary_Materials.docx]

**Supplementary Materials**

**AP1S3 Promotes Proliferation and Invasion of Cholangiocarcinoma Cells and Modulates HER2 Trafficking**

Contents: Supplementary Figures S1–S7; Supplementary Tables S1–S22; Supplementary Code S1; Supplementary Computational Note S1.

*All captions and file names are in English. Tables are also provided as CSV files with matching names.*

**List of Supplementary Items**

| **Item** | **Caption** |
| --- | --- |
| Supplementary Figure S1 | Mendelian randomization sensitivity analyses. (A) Forest plot of the strict inverse-variance-weighted (IVW) estimate (8 IVs; MHC excluded). (B) MHC-region sensitivity analysis. (C) Exploratory two-step MR mediation estimates. (D) Leave-one-out sensitivity analysis for the strict IVW estimate. |
| Supplementary Figure S2 | Secondary cis-eQTL Mendelian randomization of the nine candidate genes. Forest plots show the IVW estimates for the nine genes prioritized from the 553-gene screen. These analyses were exploratory and nominal P values were used only for prioritization. |
| Supplementary Figure S3 | Kaplan–Meier survival analysis of the nine candidate genes in the Fu-iCCA cohort. Curves compare high versus low expression for each gene. These analyses are exploratory and not adjusted for multiple testing. |
| Supplementary Figure S4 | Pan-cancer AP1S3 expression across TCGA tumour and normal tissues. Box plots show AP1S3 expression (log2 TPM) in tumour and adjacent normal samples. This comparison is descriptive. |
| Supplementary Figure S5 | AP1S3 overexpression validation in HuCCT1 and QBC939 cells. (A) Western blot and qPCR validation of AP1S3 overexpression. (B) Colony formation. (C) EdU incorporation. (D) CCK-8 proliferation. (E,F) Migration and invasion assays. Data are from three biological replicates. |
| Supplementary Figure S6 | Corrected HER2 EIPDLL motif and per-residue solvent-accessible surface area (SASA). The EIPDLL motif is at residues 930–935 within the HER2 kinase domain, not at 1001–1015 and not in the C-terminal intrinsically disordered region. SASA was calculated on HER2 kinase structure 3PP0; I931 and L935 are largely buried. Accessibility was not tested experimentally. |
| Supplementary Figure S7 | Computational proof-of-concept for targeting the candidate AP1S3–HER2 interface. (A) Virtual screening and in silico optimization workflow. (B) Docking poses and scores. (C–G) Single 100-ns MD simulations (RMSD, SASA, Rg, hydrogen bonds, RMSF). These data are computational only and are not experimental validation; the docking-score difference is within Vina error, and no replicate MD trajectories or free-energy calculations were performed. |
| Supplementary Table S1 | Mendelian randomization instrument variables. |
| Supplementary Table S2 | Mendelian randomization sensitivity analyses. |
| Supplementary Table S3 | Exploratory two-step MR mediation estimates. |
| Supplementary Table S4 | Sample overlap between exposure and outcome GWAS. |
| Supplementary Table S5 | Pre-specified confounder definitions. |
| Supplementary Table S6 | STROBE-MR checklist. |
| Supplementary Table S7 | AP1S3 cis-eQTL Mendelian randomization. |
| Supplementary Table S8 | TCGA-CHOL Cox proportional-hazards analysis. |
| Supplementary Table S9 | AP1S1, AP1S2 and AP1S3 isoform comparison. |
| Supplementary Table S10 | Multiplicity inventory for exploratory analyses. |
| Supplementary Table S11 | Selection criteria for mediators and AP1S3 prioritization. |
| Supplementary Table S12 | Cytokine dose justification. |
| Supplementary Table S13 | AP-1 complex and mutant controls. |
| Supplementary Table S14 | Single-cell annotation criteria. |
| Supplementary Table S15 | Xenograft design and reporting. |
| Supplementary Table S16 | Computational reproducibility checklist. |
| Supplementary Table S17 | Statistical reporting summary. |
| Supplementary Table S18 | Clinical cohort characteristics. |
| Supplementary Table S19 | Cell-line characteristics. |
| Supplementary Table S20 | Cell-line panel and missing models. |
| Supplementary Table S21 | Numeric ADMET predictions. |
| Supplementary Table S22 | Primers and antibodies. |
| Supplementary Code S1 | MR and single-cell analysis scripts. |
| Supplementary Computational Note S1 | Computational proof-of-concept; not experimental validation. |

**Supplementary Figure S1**

**Mendelian randomization sensitivity analyses.**

(A) Forest plot of the strict inverse-variance-weighted (IVW) estimate (8 IVs; MHC excluded). (B) MHC-region sensitivity analysis. (C) Exploratory two-step MR mediation estimates. (D) Leave-one-out sensitivity analysis for the strict IVW estimate.

**Supplementary Figure S2**

**Secondary cis-eQTL Mendelian randomization of the nine candidate genes.**

Forest plots show the IVW estimates for the nine genes prioritized from the 553-gene screen. These analyses were exploratory and nominal P values were used only for prioritization.

**Supplementary Figure S3**

**Kaplan–Meier survival analysis of the nine candidate genes in the Fu-iCCA cohort.**

Curves compare high versus low expression for each gene. These analyses are exploratory and not adjusted for multiple testing.

**Supplementary Figure S4**

**Pan-cancer AP1S3 expression across TCGA tumour and normal tissues.**

Box plots show AP1S3 expression (log2 TPM) in tumour and adjacent normal samples. This comparison is descriptive.

**Supplementary Figure S5**

**AP1S3 overexpression validation in HuCCT1 and QBC939 cells.**

(A) Western blot and qPCR validation of AP1S3 overexpression. (B) Colony formation. (C) EdU incorporation. (D) CCK-8 proliferation. (E,F) Migration and invasion assays. Data are from three biological replicates.

**Supplementary Figure S6**

**Corrected HER2 EIPDLL motif and per-residue solvent-accessible surface area (SASA).**

The EIPDLL motif is at residues 930–935 within the HER2 kinase domain, not at 1001–1015 and not in the C-terminal intrinsically disordered region. SASA was calculated on HER2 kinase structure 3PP0; I931 and L935 are largely buried. Accessibility was not tested experimentally.

**Supplementary Figure S7**

**Computational proof-of-concept for targeting the candidate AP1S3–HER2 interface.**

(A) Virtual screening and in silico optimization workflow. (B) Docking poses and scores. (C–G) Single 100-ns MD simulations (RMSD, SASA, Rg, hydrogen bonds, RMSF). These data are computational only and are not experimental validation; the docking-score difference is within Vina error, and no replicate MD trajectories or free-energy calculations were performed.

**Supplementary Table S1**

**Mendelian randomization instrument variables.**

| **SNP** | **CHR** | **POS** | **EA** | **OA** | **EAF** | **beta_exp** | **SE_exp** | **P_exp** | **F** | **beta_out** | **SE_out** | **P_out** | **Wald_OR** | **Wald_P** | **MHC** | **Used** | **Reason** |
| --- | --- | --- | --- | --- | --- | --- | --- | --- | --- | --- | --- | --- | --- | --- | --- | --- | --- |
| rs1177206 | 2 | 61075189 | T | C | 0.3616 | -0.1212 | 0.0206 | 3.65e-09 | 34.62 | -0.0038 | 0.0436 | 0.9297 | 1.032 | 0.9305 | False | Yes |  |
| rs2312786 | 3 | 101633360 | A | G | 0.2314 | 0.1403 | 0.0229 | 9.61e-10 | 37.54 | 2.00e-04 | 0.0487 | 0.9965 | 1.001 | 0.9967 | False | Yes |  |
| rs10040411 | 5 | 158812417 | T | C | 0.3683 | -0.2372 | 0.0297 | 1.45e-15 | 63.78 | -0.0025 | 0.045 | 0.9554 | 1.011 | 0.9557 | False | Yes |  |
| rs12188300 | 5 | 158829527 | T | A | 0.0849 | 0.3952 | 0.0397 | 2.57e-23 | 99.1 | 0.057 | 0.0872 | 0.5128 | 1.155 | 0.5133 | False | Yes |  |
| rs9481169 | 6 | 111929862 | T | G | 0.07761 | 0.256 | 0.0373 | 6.58e-12 | 47.1 | -0.0748 | 0.0762 | 0.3264 | 0.7466 | 0.3263 | False | Yes |  |
| rs6933987 | 6 | 138239434 | T | C | 0.2255 | 0.1258 | 0.0223 | 1.78e-08 | 31.82 | -0.0697 | 0.0522 | 0.1813 | 0.5746 | 0.1818 | False | Yes |  |
| rs2249937 | 6 | 159515309 | G | T | 0.7685 | -0.158 | 0.0233 | 1.18e-11 | 45.98 | -0.009 | 0.0529 | 0.8643 | 1.059 | 0.8649 | False | Yes |  |
| rs34725611 | 19 | 10477067 | G | A | 0.3051 | -0.1636 | 0.0225 | 3.80e-13 | 52.87 | -0.0317 | 0.0435 | 0.466 | 1.214 | 0.4662 | False | Yes |  |
| rs6672420 | 1 | 25291010 | T | A | 0.5202 | 0.1237 | 0.0201 | 7.01e-10 | 37.87 | 0.0135 | 0.0406 | 0.7402 | 1.115 | 0.7395 | False | No | Palindromic A/T SNP with MAF >0.42 (ambiguous) |
| rs3116852 | 6 | 29135647 | G | A | 0.5307 | 0.1199 | 0.021 | 1.19e-08 | 32.6 | 0.0446 | 0.041 | 0.2768 | 1.451 | 0.2767 | True | No | MHC region chr6:25-34 Mb (GRCh37) |
| rs2734967 | 6 | 29835518 | A | T | 0.3074 | 0.1451 | 0.0229 | 2.52e-10 | 40.15 | 0.0571 | 0.0446 | 0.2006 | 1.482 | 0.2005 | True | No | MHC region chr6:25-34 Mb (GRCh37) |
| rs12212594 | 6 | 31300819 | C | T | 0.05513 | 1.445 | 0.0468 | 4.82e-209 | 953.9 | 0.0694 | 0.0941 | 0.461 | 1.049 | 0.4608 | True | No | MHC region chr6:25-34 Mb (GRCh37) |
| rs68094537 | 6 | 31397828 | C | T | 0.2838 | 0.3628 | 0.024 | 1.80e-51 | 228.5 | 0.026 | 0.0469 | 0.5797 | 1.074 | 0.5793 | True | No | MHC region chr6:25-34 Mb (GRCh37) |
| rs61268343 | 6 | 32615050 | C | T | 0.07113 | 0.7148 | 0.0804 | 6.07e-19 | 79.04 | 0.1281 | 0.0888 | 0.149 | 1.196 | 0.1491 | True | No | MHC region chr6:25-34 Mb (GRCh37) |
| rs9277195 | 6 | 33023926 | T | C | 0.515 | -0.1215 | 0.0207 | 4.23e-09 | 34.45 | -0.0155 | 0.0408 | 0.7044 | 1.136 | 0.704 | True | No | MHC region chr6:25-34 Mb (GRCh37) |

**Supplementary Table S2**

**Mendelian randomization sensitivity analyses.**

| **Role** | **Analysis** | **Exposure** | **Outcome** | **Threshold** | **Clumping_window** | **MHC** | **N_IV** | **OR** | **CI_low** | **CI_high** | **P** | **Q_P** | **Egger_intercept_P** | **MR_PRESSO_P** | **PRESSO_outliers** | **Weighted_median_P** | **F_median** | **F_min** | **Detectable_OR_80pct** | **Power_at_OR_1.12** | **Egger_OR** | **WM_OR** | **Egger_slope_P** |
| --- | --- | --- | --- | --- | --- | --- | --- | --- | --- | --- | --- | --- | --- | --- | --- | --- | --- | --- | --- | --- | --- | --- | --- |
| Main | Primary: P<5e-8, MHC excluded | Sakaue 2021 (GCST90018907) | Sakaue 2021 CCA (GCST90018803) | P<5e-8 | 10,000 kb | Excluded | 8 | 1.002 | 0.827 | 1.214 | 0.9811 | 0.8088 | 0.5399 | 0.7967 | 0 | 0.8814 | 46.5 | 31.8 | 1.315 | 0.211 | 1.135 | 1.026 | 0.558 |
| Sensitivity | Sensitivity: 1,000 kb clumping window | Sakaue 2021 (GCST90018907) | Sakaue 2021 CCA (GCST90018803) | P<5e-8 | 1,000 kb | Excluded | 9 | 0.947 | 0.793 | 1.131 | 0.5461 | 0.6338 | 0.8829 | 0.6333 | 0 | 0.9272 | 47.1 | 31.8 | 1.289 | 0.239 | 0.917 | 1.016 | 0.71 |
| Sensitivity | Sensitivity: 250 kb clumping window | Sakaue 2021 (GCST90018907) | Sakaue 2021 CCA (GCST90018803) | P<5e-8 | 250 kb | Excluded | 9 | 0.947 | 0.793 | 1.131 | 0.5461 | 0.6338 | 0.8829 | 0.6333 | 0 | 0.9272 | 47.1 | 31.8 | 1.289 | 0.239 | 0.917 | 1.016 | 0.71 |
| Reconstructed original | Primary re-analysis: P<1e-5, MHC excluded | Sakaue 2021 (GCST90018907) | Sakaue 2021 CCA (GCST90018803) | P<1e-5 | 10,000 kb | Excluded | 50 | 1.125 | 1.005 | 1.26 | 0.04113 | 0.3155 | 0.5007 | 0.31 | 1 | 0.925 | 24.5 | 19.6 | 1.175 | 0.502 | 1.233 | 1.035 | 0.157 |
| Sensitivity | Sensitivity: MHC included | Sakaue 2021 (GCST90018907) | Sakaue 2021 CCA (GCST90018803) | P<5e-8 | 10,000 kb | Included | 14 | 1.076 | 0.985 | 1.175 | 0.1055 | 0.901 | 0.8368 | 0.9233 | 0 | 0.7318 | 46.5 | 31.8 | 1.134 | 0.712 | 1.068 | 1.054 | 0.18 |
| Replication | Independent exposure: strict, MHC excluded | Stuart 2021 (GCST90019016) | Sakaue 2021 CCA (GCST90018803) | P<5e-8 | 10,000 kb | Excluded | 49 | 1.047 | 0.959 | 1.144 | 0.3044 | 0.4743 | 0.7264 | 0.4667 | 1 | 0.871 | 47.7 | 30.6 | 1.135 | 0.712 | 1.082 | 1.047 | 0.447 |
| Replication | Independent exposure: 1,000 kb | Stuart 2021 (GCST90019016) | Sakaue 2021 CCA (GCST90018803) | P<5e-8 | 1,000 kb | Excluded | 50 | 1.025 | 0.939 | 1.119 | 0.583 | 0.3682 | 0.9935 | 0.3967 | 2 | 0.8808 | 46 | 30.6 | 1.134 | 0.717 | 1.026 | 1.042 | 0.801 |
| Replication | Independent exposure: 250 kb | Stuart 2021 (GCST90019016) | Sakaue 2021 CCA (GCST90018803) | P<5e-8 | 250 kb | Excluded | 58 | 0.998 | 0.918 | 1.084 | 0.9553 | 0.3666 | 0.9004 | 0.39 | 2 | 0.9646 | 45.8 | 30.6 | 1.125 | 0.762 | 1.008 | 1.012 | 0.932 |
| Replication | Independent exposure: P<1e-5 | Stuart 2021 (GCST90019016) | Sakaue 2021 CCA (GCST90018803) | P<1e-5 | 10,000 kb | Excluded | 96 | 1.009 | 0.935 | 1.089 | 0.8141 | 0.4999 | 0.7419 | 0.52 | 5 | 0.8953 | 30.6 | 19.7 | 1.115 | 0.83 | 1.036 | 1.045 | 0.69 |
| Replication | Independent exposure: MHC included | Stuart 2021 (GCST90019016) | Sakaue 2021 CCA (GCST90018803) | P<5e-8 | 10,000 kb | Included | 55 | 1.067 | 0.994 | 1.146 | 0.07309 | 0.6172 | 0.4684 | 0.65 | 1 | 0.7436 | 50.3 | 30.6 | 1.107 | 0.877 | 1.109 | 1.084 | 0.103 |
| Reverse | Reverse MR: CCA to psoriasis (Sakaue outcome) | Sakaue 2021 CCA (GCST90018803) | Sakaue 2021 psoriasis | P<1e-5 | 10,000 kb | Excluded | 19 | 1.014 | 0.979 | 1.051 | 0.4306 | 0.6859 | 0.5249 | 0.7067 | 3 | 0.7386 | 21.2 | 19.6 | 1.052 | 1 | 1.027 | 1.02 | 0.3 |
| Reverse | Reverse MR: CCA to psoriasis (Stuart outcome) | Stuart 2021 (GCST90019016) | Stuart 2021 psoriasis | P<1e-5 | 10,000 kb | Excluded | 15 | 0.996 | 0.967 | 1.026 | 0.7969 | 0.5663 | 0.6727 | 0.5867 | 0 | 0.9141 | 20.7 | 19.7 | 1.044 | 1 | 0.99 | 0.994 | 0.63 |
| Sensitivity | Sensitivity: IL12B/TYK2 loci removed | Sakaue 2021 (GCST90018907) | Sakaue 2021 CCA (GCST90018803) | P<5e-8 | 10,000 kb | Excluded | 5 | 0.872 | 0.645 | 1.18 | 0.375 |  |  |  |  |  |  |  |  | 0.11 |  |  |  |

**Supplementary Table S3**

**Exploratory two-step MR mediation estimates.**

| **Mediator** | **Mediator_GWAS** | **Path_a_beta** | **Path_a_SE** | **Path_b_beta** | **Path_b_SE** | **Total_c_beta** | **Total_c_SE** | **Indirect_a_x_b** | **Indirect_SE** | **Indirect_95CI** | **Sobel_P** | **Proportion_mediated_pct** | **Proportion_95CI_conditional_pct** | **Direction** | **Interpretation** |
| --- | --- | --- | --- | --- | --- | --- | --- | --- | --- | --- | --- | --- | --- | --- | --- |
| GPE (p-16:0/18:2) | GCST90300318 | -0.02802 | 0.01362 | -0.4475 | 0.161 | 0.1165 | 0.05583 | 0.01254 | 0.007582 | -0.002 to 0.027 | 0.09822 | 10.76 | -2.4 to 66.6 | a<0, b<0; lower mediator -> higher CCA risk | 95% CI includes zero; exploratory only |
| CD127 on CD8+ T cell | GCST90001929 | -0.05871 | 0.02914 | -0.3415 | 0.1626 | 0.1165 | 0.05583 | 0.02005 | 0.01379 | -0.007 to 0.047 | 0.146 | 17.21 | -6.3 to 108.6 | a<0, b<0; lower mediator -> higher CCA risk | 95% CI includes zero; exploratory only |
| CD4-CD8- T cell % | GCST90001613 | -0.05411 | 0.02629 | -0.3608 | 0.1643 | 0.1165 | 0.05583 | 0.01952 | 0.013 | -0.006 to 0.045 | 0.1331 | 16.76 | -5.0 to 107.3 | a<0, b<0; lower mediator -> higher CCA risk | 95% CI includes zero; exploratory only |

**Supplementary Table S4**

**Sample overlap between exposure and outcome GWAS.**

| **Item** | **Value** |
| --- | --- |
| Psoriasis GWAS | Sakaue et al. 2021, GCST90018907; 5,072 European cases/478,102 controls; 206 East Asian cases/172,289 controls; cohorts BBJ, UKB, FinnGen |
| CCA GWAS | Sakaue et al. 2021, GCST90018803; 832 European cases/475,259 controls; 418 East Asian cases/159,201 controls; cohorts BBJ, UKB, FinnGen |
| Shared cohorts | Both traits were meta-analysed from the same three cohorts (BBJ, UKB, FinnGen). |
| Maximum shared controls, European | 475,259 (100% of CCA controls; 99.4% of psoriasis controls) |
| Maximum shared controls, East Asian | 159,201 (100% of CCA controls; 92.4% of psoriasis controls) |
| Case overlap | Different diseases; overlap expected to be small but not zero (individuals with both conditions). |
| Empirical check | Cross-trait LDSC intercept = 0.003 (SE 0.0043; P=0.485); genetic correlation = 0.284 (SE 0.248; P=0.252). |
| Independent replication | Stuart et al. 2021 (GCST90019016; 15,967 European cases/28,194 controls; CASP, Kiel, Genizon, PsA, WTCCC2, Exomechip, PAGE, GAPC) has no cohort overlap with UKB, FinnGen or BBJ. |
| Replication result | IVW OR 1.047 (95% CI 0.959-1.144), P=0.304; MHC-included OR 1.067 (0.994-1.146), P=0.073. |

**Supplementary Table S5**

**Pre-specified confounder definitions.**

| **Trait_domain** | **Rationale** | **Action_in_revised_analysis** |
| --- | --- | --- |
| Smoking initiation / current smoking | Established CCA and psoriasis risk factor; potential confounder | Listed a priori; not used for unstructured filtering |
| Alcohol intake / alcohol use disorder | Established CCA risk factor | Listed a priori; not used for unstructured filtering |
| Body mass index / obesity | Established CCA risk factor; shared with psoriasis | Listed a priori; not used for unstructured filtering |
| Type 2 diabetes / fasting glucose | Established CCA risk factor | Listed a priori; not used for unstructured filtering |
| Inflammatory bowel disease (Crohn disease, ulcerative colitis) | Established PSC/CCA pathway; shared autoimmune architecture | Listed a priori; not used for unstructured filtering |
| Primary sclerosing cholangitis | Strongest CCA risk factor | Listed a priori; not used for unstructured filtering |
| Hepatitis B / hepatitis C | Established CCA risk factors | Listed a priori; not used for unstructured filtering |
| Cirrhosis / NAFLD / alcohol-related liver disease | Established CCA risk factors | Listed a priori; not used for unstructured filtering |
| Psoriasis, psoriatic arthritis, ankylosing spondylitis, rheumatoid arthritis | Autoimmune traits that index shared pleiotropy | Listed a priori; global pleiotropy tested by MR-Egger/MR-PRESSO |
| Age, sex, ancestry | Demographic confounders | Adjusted by study design/ancestry restriction; not SNP-level traits |

**Supplementary Table S6**

**STROBE-MR checklist.**

| **Item** | **Topic** | **Requirement** | **Where_addressed** |
| --- | --- | --- | --- |
| 1 | Title and abstract | State that MR was used and summarize the causal question | Abstract; Methods 1.1 |
| 2 | Background/rationale | Explain rationale for psoriasis-CCA MR | Introduction |
| 3 | Objectives | Test whether genetic liability to psoriasis affects CCA risk | Methods 1.1 |
| 4 | Study design | Two-sample MR; forward and reverse | Methods 1.1 |
| 5 | Data sources | GWAS Catalog IDs, ancestry, sample sizes, overlap | Methods 1.1; Table S4 |
| 6 | Instrument selection | P<5e-8; LD r2<0.001; 10,000 kb; MHC excluded; F>10 | Methods 1.2; Table S1 |
| 7 | Power | Report detectable OR and post-hoc power | Methods 1.6; Results 2.1 |
| 8 | Statistical methods | IVW, weighted median, MR-Egger, MR-PRESSO, Q, Steiger | Methods 1.3 |
| 9 | Pleiotropy | MHC exclusion; Egger intercept; MR-PRESSO; IL12B/TYK2 sensitivity | Methods 1.4; Table S2 |
| 10 | Reverse direction | CCA to psoriasis MR | Methods 1.5; Table S2 |
| 11 | Mediation | Two-step MR; proportion and 95% CI; exploratory only | Methods 1.7; Table S3 |
| 12 | Main results | Report strict and relaxed estimates; do not overstate | Results 2.1; Figure S1 |
| 13 | Sensitivity | Clumping window, MHC, independent exposure | Results 2.1; Table S2 |
| 14 | Limitations | Low power; overlapping controls; weak mediator instruments | Discussion |
| 15 | Data/code | Public GWAS; analysis code available | Data Availability |

**Supplementary Table S7**

**AP1S3 cis-eQTL Mendelian randomization.**

| **Analysis** | **SNP** | **EA** | **OA** | **EAF** | **beta_exposure** | **SE_exposure** | **P_exposure** | **F** | **beta_outcome** | **SE_outcome** | **P_outcome** | **OR** | **CI_low** | **CI_high** | **P** |
| --- | --- | --- | --- | --- | --- | --- | --- | --- | --- | --- | --- | --- | --- | --- | --- |
| Single SNP | rs10209883 | A | G | 0.75 | 0.0566 | 0.0092 | 8.87e-10 | 37.56 | -0.0418 | 0.056 | 0.4555 | 0.478 | 0.0688 | 3.321 | 0.4554 |
| Single SNP | rs116000059 | A | C | 0.0248 | 0.1508 | 0.0267 | 1.59e-08 | 31.94 | 0.1131 | 0.1399 | 0.4189 | 2.117 | 0.3436 | 13.04 | 0.4188 |
| Single SNP | rs116476702 | T | C | 0.0239 | 0.2371 | 0.0422 | 1.93e-08 | 31.57 | 0.0272 | 0.1841 | 0.8825 | 1.122 | 0.2448 | 5.138 | 0.8825 |
| Single SNP | rs13001654 | T | C | 0.1171 | -0.0845 | 0.0124 | 1.10e-11 | 46.14 | -0.1199 | 0.0714 | 0.093 | 4.131 | 0.7889 | 21.63 | 0.0931 |
| Single SNP | rs6436440 | A | G | 0.5225 | 0.1847 | 0.008 | 2.85e-119 | 539.2 | 0.084 | 0.0403 | 0.0372 | 1.576 | 1.028 | 2.417 | 0.0371 |
| Single SNP | rs7606306 | T | C | 0.3626 | -0.0574 | 0.0084 | 6.60e-12 | 47.14 | -0.0475 | 0.0443 | 0.2838 | 2.286 | 0.5043 | 10.36 | 0.2836 |
| Single SNP | rs79305596 | T | C | 0.0876 | -0.0989 | 0.0142 | 2.75e-12 | 48.85 | 0.0419 | 0.0804 | 0.6021 | 0.6547 | 0.1331 | 3.22 | 0.6023 |
| IVW (7 SNPs) |  |  |  |  |  |  |  |  |  |  |  | 1.534 | 1.069 | 2.201 | 0.0203 |
| Weighted median |  |  |  |  |  |  |  |  |  |  |  | 1.574 | 1.06 | 2.337 | 0.0244 |
| MR-Egger |  |  |  |  |  |  |  |  |  |  |  | 1.604 | 0.7419 | 3.469 | 0.2834 |
| MR-PRESSO global test |  |  |  |  |  |  |  |  |  |  |  |  |  |  | 0.628 |

**Supplementary Table S8**

**TCGA-CHOL Cox proportional-hazards analysis.**

| **Model** | **N** | **Events** | **Term** | **HR** | **CI_low** | **CI_high** | **P** | **LR_interaction_P** |
| --- | --- | --- | --- | --- | --- | --- | --- | --- |
| Unadjusted | 33 | 18 | AP1S3_z | 0.8896 | 0.5487 | 1.442 | 0.6352 |  |
| Unadjusted | 33 | 18 | ERBB2_z | 1.636 | 0.6361 | 4.21 | 0.307 |  |
| Unadjusted | 33 | 18 | AP1S3_z:ERBB2_z | 2.108 | 0.952 | 4.668 | 0.06595 | 0.0285 |
| Age + sex | 33 | 18 | AP1S3_z | 0.7749 | 0.4444 | 1.351 | 0.3685 |  |
| Age + sex | 33 | 18 | ERBB2_z | 1.731 | 0.6449 | 4.648 | 0.276 |  |
| Age + sex | 33 | 18 | AP1S3_z:ERBB2_z | 2.198 | 0.927 | 5.212 | 0.07379 | 0.03089 |
| Age + sex + stage | 23 | 8 | AP1S3_z | 0.8313 | 0.2913 | 2.372 | 0.7298 |  |
| Age + sex + stage | 23 | 8 | ERBB2_z | 1.473 | 0.5441 | 3.985 | 0.4461 |  |
| Age + sex + stage | 23 | 8 | AP1S3_z:ERBB2_z | 1.623 | 0.6326 | 4.163 | 0.3138 | 0.2304 |
| Subgroup: ERBB2-low (continuous) | 17 | 10 | AP1S3_z | 0.5768 | 0.2398 | 1.388 | 0.2192 |  |
| Subgroup: ERBB2-high (continuous) | 16 | 8 | AP1S3_z | 1.563 | 0.6268 | 3.899 | 0.338 |  |
| Log-rank: ERBB2-low | 17 | 10 | AP1S3 high vs low |  |  |  | 0.3415 |  |
| Log-rank: ERBB2-high | 16 | 8 | AP1S3 high vs low |  |  |  | 0.122 |  |

**Supplementary Table S9**

**AP1S1, AP1S2 and AP1S3 isoform comparison.**

| **Isoform** | **UniProt** | **Length** | **Identity_to_AP1S3_pct** | **TCGA_CHOL_median_log2** | **Fu_iCCA_median_log2** |
| --- | --- | --- | --- | --- | --- |
| AP1S1 (σ1A) | P61966 | 158 | 69.5 | 11.35 | 4.95 |
| AP1S2 (σ1B) | P56377 | 157 | 73.2 | 9.28 | 5.3 |
| AP1S3 (σ1C) | Q96PC3 | 154 | 100 | 8.58 | 4.37 |

**Supplementary Table S10**

**Multiplicity inventory for exploratory analyses.**

| **Analysis** | **Number_of_tests** | **Correction** | **Threshold_or_q** | **Result** | **Interpretation** |
| --- | --- | --- | --- | --- | --- |
| Primary psoriasis→CCA (strict) | 1 | None (single pre-specified hypothesis) | 0.05 | IVW OR 1.002 (0.827–1.214); P=0.981 | Inconclusive; no causal claim |
| Original relaxed P<1e-5 | 1 | Bonferroni across 659 exploratory tests | 7.6×10⁻⁵ | IVW OR 1.125 (1.005–1.260); P=0.041 | Would not survive correction |
| Secondary cis-eQTL MR (553 genes) | 553 | Bonferroni / nominal prioritization | 9.0×10⁻⁵ | AP1S3 P=0.020; rs6436440-driven | Not significant after correction; candidate only |
| Mediation (3 mediators) | 3 | Benjamini–Hochberg FDR | q=0.146 | All Sobel P>0.09; all CIs include zero | Not significant |
| Sensitivity analyses (MHC, window, independent exposure, reverse) | — | Not independent primary tests | — | Strict OR 1.002; independent OR 1.047; reverse OR 1.014 | Support inconclusive interpretation |

**Supplementary Table S11**

**Selection criteria for mediators and AP1S3 prioritization.**

| **Step** | **Criterion** | **Number_retained** | **Threshold** | **Interpretation** |
| --- | --- | --- | --- | --- |
| Step 1: DEG intersection | GSE54456 psoriasis lesion vs normal; TCGA-CHOL tumour vs normal; log2FC>1 and FDR<0.05 in both | 553 | FDR<0.05 | Discovery set; not a mediation test |
| Step 2: secondary cis-eQTL MR | eQTLGen cis-eQTL instruments; nominal P<0.05 for prioritization | 9 | P<0.05 (nominal) | Exploratory; no FDR control |
| Step 3: survival filter | Fu-iCCA cohort; P<0.05 and HR>1 | 1 (AP1S3) | P<0.05 | Data-driven sequential filter; independent cohort but selection bias possible |
| Step 4: AP1S3 cis-eQTL MR | 7 SNPs; IVW OR 1.53 (1.07–2.20), P=0.020 | 1 | Bonferroni 9 genes: 0.0056; 553 genes: 9.0×10⁻⁵ | Not significant after correction; candidate only |
| Mediator screen | Path a and path b both nominal P<0.05; 3 mediators | 3 | P<0.05 | BH-FDR q=0.146; all indirect CIs include zero |

**Supplementary Table S12**

**Cytokine dose justification.**

| **Agent** | **Dose_used** | **Typical_circulating_level** | **Approx_fold_above_serum** | **Rationale** | **Limitation** |
| --- | --- | --- | --- | --- | --- |
| IL-17A | 100 ng/mL | Low pg/mL to tens of pg/mL in serum | ~10³–10⁴-fold | Prior mechanistic studies in CCA/keratinocytes; robust NF-κB activation in 24–48 h | Supraphysiologic; no dose-response; no single-cytokine control |
| TNF-α | 50 ng/mL | Low pg/mL to tens of pg/mL in serum | ~10²–10³-fold | Same as above | Supraphysiologic; combination may activate multiple pathways |
| IL-17A + TNF-α | 100 + 50 ng/mL | — | — | Synergistic NF-κB activation | Cannot attribute effect to one cytokine; no physiological validation |
| Lower doses / patient fluids | 0.1–10 ng/mL; 5–10% serum/conditioned medium | — | — | Proposed validation | Not performed in this revision |

**Supplementary Table S13**

**AP-1 complex and mutant controls.**

| **Control_experiment** | **Design** | **Purpose** |
| --- | --- | --- |
| AP1S3 vs AP1S1/AP1S2 | Separate knockdown/rescue of each σ1 isoform; HER2 surface/recycling | Test isoform specificity |
| AP-1 complex | AP-1 γ/β knockdown; native PAGE/Co-IP for complex integrity | Test hemicomplex requirement |
| Mutant control | EIPDAA vs WT EIPDLL vs control mutation outside motif | Exclude general folding effect |
| AP1S3-null background | HER2-WT vs HER2-EIPDAA in AP1S3 knockout; surface/total/degradation/phosphorylation | Directly test motif dependence |
| Localization | HER2-GFP with LAMP1, EEA1, Rab4, Rab11, CD63; live imaging/quantitative colocalization | Distinguish lysosome vs recycling endosome |
| Ubiquitination | HER2 K48/K63 ubiquitination ± AP1S3; proteasome/lysosome inhibitors | Define sorting mechanism |
| Surface/recycling controls | Internalization, MesNa efficiency, total surface normalization | Make recycling rate interpretable |

**Supplementary Table S14**

**Single-cell annotation criteria.**

| **Lineage_or_subtype** | **Markers_or_method** | **Definition** | **Validation_status** |
| --- | --- | --- | --- |
| Epithelial | EPCAM, KRT19, KRT8, KRT18 | Canonical epithelial markers | Marker-based; no pathology review |
| Immune | PTPRC, CD3D, CD68 | Pan-immune/T/NK/myeloid | Marker-based |
| Fibroblast | COL1A1, COL3A1, DCN | Fibroblast/CAF | Marker-based |
| Endothelial | PECAM1, VWF | Endothelial | Marker-based |
| Malignant epithelial | Epithelial markers + inferCNV | CNV inference | Computational; no FISH/pathology confirmation |
| SCEL+ squamous | SCEL, KRT13, KRT16, KRT6A | Squamous/keratinization program | Cluster association; not a validated subtype |
| Independent validation | TCGA-CHOL and Fu-iCCA bulk expression/survival | Independent cohorts for association | Bulk, not single-cell/IHC; no direct validation |

**Supplementary Table S15**

**Xenograft design and reporting.**

| **Item** | **Design_or_status** | **Limitation** |
| --- | --- | --- |
| Model | HuCCT1 subcutaneous xenograft; 4-week-old female BALB/c nude mice | Immunodeficient; no systemic psoriasis inflammation |
| Groups | shNC; shAP1S3; shAP1S3 + Flag-HER2 | 3 groups |
| Animal numbers | n=5 per group; 15 total | No a priori power calculation |
| Allocation | Grouped after tumour formation; randomization method not documented | Selection bias possible |
| Blinding | Tumour measurement and analysis not documented as blinded | Detection bias possible |
| HER2 reconstitution | Exogenous Flag-HER2 overexpression; not CRISPR knock-in; not titrated to endogenous level | May exceed physiological HER2; rescue = compensation |
| Tumour volume | Measured every 3 days; V=0.5×length×width² | Two-way ANOVA (time×group) with Bonferroni in Methods |
| Endpoint | Tumour weight after sacrifice; IF for HER2/p-HER2 | One-way ANOVA/Tukey in Methods |
| Limitations | One cell line; subcutaneous; no orthotopic/immunocompetent model | Does not model psoriasis systemic inflammation |

**Supplementary Table S16**

**Computational reproducibility checklist.**

| **Item** | **Available_or_status** | **Missing_or_limitation** |
| --- | --- | --- |
| Compound identity | STK040969 and STK040969-opt; SMILES printed in ADMETlab reports | SMILES not independently verified; no atom-level mapping to optimization steps |
| Chemical synthesis | Not performed | No purity, NMR, MS or batch record |
| Receptor structure | AP1S3 model used for docking; PDB/model source not recorded | Receptor preparation/protonation not documented |
| Docking | DrugCLIP and Vina scores; -6.48 vs -7.17 kcal/mol | Docking box, exhaustiveness, protonation and software versions not fully recorded; 0.7 kcal/mol within Vina error |
| MD | GROMACS 2022; one 100 ns trajectory per complex | Force field, water/ions, thermostat/barostat, seeds and replicates not recorded |
| Free energy | Not performed | No MM-PBSA/GBSA or absolute binding free energy |
| ADMET | ADMETlab 3.0 predictions | ML predictions; no experimental solubility, PPB, CYP, hERG or Caco-2 |
| Pocket validation |  | No mutagenesis or EIPDLL competition |
| Selectivity |  | AP-1 is a shared adaptor; broad off-target trafficking possible |
| Experimental validation |  | No synthesis, SPR/ITC, cellular activity or PK |

**Supplementary Table S17**

**Statistical reporting summary.**

| **Analysis** | **n** | **Effect_size (95% CI)** | **Exact_P** | **Test** | **Multiplicity** |
| --- | --- | --- | --- | --- | --- |
| MR strict (psoriasis→CCA) | 8 IVs | OR 1.002 (95% CI 0.827–1.214) | P=0.981 | IVW; F median 46.5, min 31.8 | Single pre-specified test; no correction |
| MR original P<1e-5 | 50 IVs | OR 1.125 (95% CI 1.005–1.260) | P=0.041 | IVW | Would not survive 659-test Bonferroni (7.6e-5) |
| MR independent exposure | 49 IVs | OR 1.047 (95% CI 0.959–1.144) | P=0.304 | IVW | Replication; no correction |
| Reverse MR | 19 IVs | OR 1.014 (95% CI 0.979–1.051) | P=0.431 | IVW | Underpowered |
| AP1S3 cis-eQTL MR | 7 SNPs | OR 1.534 (95% CI 1.069–2.201) | P=0.020 | IVW; F median 46.1, min 31.6 | Fails Bonferroni 9/553 genes |
| Mediation GPE | — | 10.8% (95% CI −2.4 to 66.6) | Sobel P=0.098 | Two-step MR; a×b/c | BH q=0.146 |
| Mediation CD127 on CD8+ T | — | 17.2% (95% CI −6.3 to 108.6) | Sobel P=0.146 | Two-step MR | BH q=0.146 |
| Mediation CD4−CD8− T | — | 16.8% (95% CI −5.0 to 107.3) | Sobel P=0.133 | Two-step MR | BH q=0.146 |
| TCGA-CHOL AP1S3 Cox | 33 tumours; 18 deaths | HR 0.83 (95% CI 0.29–2.37) | P=0.73 | Cox proportional hazards | Exploratory; no correction |
| Figure 5A HER2-high log-rank | 33 tumours | — | P=0.12 | Kaplan–Meier/log-rank | Subgroup; interaction P=0.066 unadjusted, 0.314 stage-adjusted |
| Figure 5A HER2-low log-rank | 33 tumours | — | P=0.34 | Kaplan–Meier/log-rank | Subgroup |
| Figure 1I vascular invasion | 7 paired cases | — | P=0.052 | Paired test | Descriptive; not adjusted |
| Figure 2 knockdown panels | 3 biological replicates | Mean differences in Prism source files | Exact P in Prism source files | One-way ANOVA + Dunnett vs shared shNC | Dunnett correction for 2 shAP1S3 groups |
| CCK-8 (Figures 2E, 5C) | 3 biological; 5 technical wells per experiment | Technical wells averaged before analysis | Exact P in Prism source files | One-way/two-way ANOVA | Technical wells not counted as n |
| Xenograft growth (Figure 5E) | 5 mice/group; 3 groups | Growth curves in Prism source files | Exact P in Prism source files | Two-way repeated-measures ANOVA (time × group; mouse as subject) | Endpoint tumour weight secondary one-way ANOVA |
| Other in vitro panels | n=3 biological unless stated | Mean differences in Prism source files | Exact P in Prism source files | t-test/ANOVA | Tukey/Bonferroni; asterisks in figures |

**Supplementary Table S18**

**Clinical cohort characteristics.**

| **Characteristic** | **Value** | **Note** |
| --- | --- | --- |
| Cohort size | 7 patients | Paired tumour and adjacent normal tissue |
| Centre | Single centre (Second Xiangya Hospital) | Convenience sample; consecutive enrollment not recorded |
| Diagnosis | Pathologically confirmed CCA | All 7 cases |
| Specimen | Paired tumour/adjacent normal | Adjacent normal distance/matching not recorded |
| Age | Not available in de-identified dataset | Cannot assess age distribution |
| Sex | Not available | Cannot assess sex distribution |
| Tumour location | Not available | Intrahepatic vs extrahepatic not recorded |
| Histology/differentiation | Not available | Subtype/grade not recorded |
| TNM stage | Not available | Stage distribution unknown |
| HBV/HCV/cirrhosis | Not available | Confounding not assessable |
| Psoriasis history | Not available | No psoriasis subgroup |
| Power | No a priori calculation | Descriptive only; underpowered for subgroup analyses |
| Analysis | Paired descriptive comparison | No adjustment for confounders |

**Supplementary Table S19**

**Cell-line characteristics.**

| **Cell_line** | **Origin** | **Source** | **Authentication** | **AP1S3** | **HER2_protein** | **HER2_amplification** | **Rationale** | **Limitation** |
| --- | --- | --- | --- | --- | --- | --- | --- | --- |
| HuCCT1 | Human intrahepatic cholangiocarcinoma | Cell Bank of Chinese Academy of Sciences | STR-authenticated; mycoplasma-free | Detectable; knockdown validated | Detectable by Western blot (Figure 4L) | Not determined (no FISH/qPCR/CNV) | Established CCA line; lentivirally transducible; intrahepatic background | HER2 expression not quantified; amplification unknown |
| QBC939 | Human bile duct cholangiocarcinoma | Guangzhou Jennio Biotech | STR-authenticated; mycoplasma-free | Detectable; knockdown validated | Detectable by Western blot (Figure 4L) | Not determined (no FISH/qPCR/CNV) | Second CCA line; independent genetic background | HER2 expression not quantified; amplification unknown |
| 293T | Human embryonic kidney | Not a CCA line | STR-authenticated; mycoplasma-free | Not applicable | Not applicable | Not applicable | Lentiviral packaging and Co-IP only | Not a CCA model; not used for HER2 status |

**Supplementary Table S20**

**Cell-line panel and missing models.**

| **Cell_model** | **Origin** | **Source_or_status** | **Used_in_study** | **HER2_status** | **Rationale_or_gap** |
| --- | --- | --- | --- | --- | --- |
| HuCCT1 | Intrahepatic CCA | Cell Bank of Chinese Academy of Sciences, Shanghai | Used | HER2 protein detectable; amplification not determined | Established intrahepatic model; lentivirally transducible |
| QBC939 | Bile duct/extrahepatic CCA | Guangzhou Jennio Biotech, Guangzhou | Used | HER2 protein detectable; amplification not determined | Second CCA background; not equivalent to HuCCT1 |
| 293T | Human embryonic kidney | Guangzhou Jennio Biotech, Guangzhou | Used for packaging/Co-IP | Not applicable | Not a CCA model |
| H69 | Non-malignant human cholangiocyte | Not used | Not used | Not determined | Would provide non-malignant control |
| HIBEC | Human intrahepatic biliary epithelial cells | Not used | Not used | Not determined | Would provide non-malignant control |
| RBE | Human intrahepatic CCA | Not used | Not used | Not determined | Additional CCA line |
| HuH28 | Human extrahepatic CCA | Not used | Not used | Not determined | Additional CCA line |
| TFK-1 | Human extrahepatic CCA | Not used | Not used | Not determined | Additional CCA line |
| Patient-derived organoids | Primary CCA | Not used | Not used | Not determined | Would improve clinical relevance |

**Supplementary Table S21**

**Numeric ADMET predictions.**

| **Property** | **STK040969** | **STK040969_opt** | **Method** | **Caveat** |
| --- | --- | --- | --- | --- |
| Molecular weight | 504.20 | 434.23 | ADMETlab 3.0 | Predicted; no experimental validation |
| logP | 3.76 | 3.24 | ADMETlab 3.0 | Predicted |
| logS | -5.688 | -3.972 | ADMETlab 3.0 | Predicted; higher logS = more soluble |
| TPSA (Å²) | 127.35 | 83.03 | ADMETlab 3.0 | Predicted |
| H-bond donors | 3 | 2 | ADMETlab 3.0 | Predicted |
| H-bond acceptors | 10 | 7 | ADMETlab 3.0 | Predicted |
| Rotatable bonds | 13 | 9 | ADMETlab 3.0 | Predicted |
| Plasma protein binding (%) | 98.1 | 87.8 | ADMETlab 3.0 / manuscript | Predicted |
| Free fraction (%) | 1.9 | 12.2 | Calculated from PPB | Predicted |
| Caco-2 permeability | Not reported numerically | Not reported numerically | ADMETlab 3.0 | Predicted; direction only |
| hERG toxicity | Not reported numerically | Not reported numerically | ADMETlab 3.0 | Predicted; direction only |
| CYP inhibition | Not reported numerically | Not reported numerically | ADMETlab 3.0 | Predicted; direction only |

**Supplementary Table S22**

**Primers and antibodies.**

| **Reagent** | **Vendor** | **Application** | **Caveat** |
| --- | --- | --- | --- |
| AP1S3 antibody | Proteintech | WB, IF | 17365-1-AP |
| HER2 antibody | Proteintech | WB, IF | 18299-1-AP |
| p-HER2 antibody | Proteintech | WB, IF | 82762-11-PBS |
| β-actin antibody | Proteintech | WB | 66009-1-Ig |
| GAPDH antibody | Proteintech | WB | 66004-1-Ig |
| AP1S3 primers | Sangon Biotech | RT-qPCR | AP1S3-F:TGGAAATGTCTGTGAGCTGGA;AP1S3-R:GGATGGGAGGCGTTGCTTAT |
| GAPDH primers | Sangon Biotech | RT-qPCR | GAPDH-F:AGAAAAACCTGCCAAATATGATGAC;GAPDH-R:TGGGTGTCGCTGTTGAAGTC |

**Supplementary Computational Note S1**

This note describes the computational proof-of-concept for targeting the candidate AP1S3–HER2 interface. It is not experimental validation.

Virtual screening used DrugCLIP, followed by structure-guided optimization. Docking scores were −6.48 kcal/mol for STK040969 and −7.17 kcal/mol for STK040969-opt; the 0.7 kcal/mol difference is within Vina scoring error. A single 100-ns MD trajectory per complex was run in GROMACS 2022; no replicates or MM-PBSA/GBSA calculations were performed. ADMET values are ADMETlab 3.0 predictions, not experimental measurements.

The compound has not been synthesized and has not been tested by SPR, ITC, pull-down, cellular assays or pharmacokinetics. The binding pocket was not validated by mutagenesis, and competition with the HER2 EIPDLL motif was not demonstrated. AP-1 is a shared adaptor, so broad off-target trafficking effects are possible.

See Supplementary Figure S7 and Supplementary Table S21 for the computational panels and numeric ADMET values. The ADMETlab reports are provided as supplementary files.

**Supplementary Code S1**

The following English-named scripts are provided in Supplementary_Code_S1.zip:

- mr_lib.py
- plot_forest.R
- plot_mhc.R
- MR_analysis.R
- single_cell_analysis_wrapper.R
- single_cell_gene_plots.R

*File inventory: Supplementary_Figure_S1.png–S7.png; Supplementary_Table_S1.csv–S22.csv; Supplementary_Code_S1.zip; Supplementary_Materials.docx.*
